# Realistic sampling of amino acid geometries for a multipolar polarizable force field

**DOI:** 10.1002/jcc.24006

**Published:** 2015-08-03

**Authors:** Timothy J. Hughes, Salvatore Cardamone, Paul L. A. Popelier

**Affiliations:** ^1^ Manchester Institute of Biotechnology (MIB)131 Princess StreetManchesterM1 7DNGreat Britain; ^2^ School of ChemistryUniversity of ManchesterOxford RoadManchesterM13 9PLGreat Britain

**Keywords:** quantum theory of atoms in molecules, quantum chemical topology, conformational sampling, kriging, electrostatics, protein data bank

## Abstract

The Quantum Chemical Topological Force Field (QCTFF) uses the machine learning method kriging to map atomic multipole moments to the coordinates of all atoms in the molecular system. It is important that kriging operates on relevant and realistic training sets of molecular geometries. Therefore, we sampled single amino acid geometries directly from protein crystal structures stored in the Protein Databank (PDB). This sampling enhances the conformational realism (in terms of dihedral angles) of the training geometries. However, these geometries can be fraught with inaccurate bond lengths and valence angles due to artefacts of the refinement process of the X‐ray diffraction patterns, combined with experimentally invisible hydrogen atoms. This is why we developed a hybrid PDB/nonstationary normal modes (NM) sampling approach called PDB/NM. This method is superior over standard NM sampling, which captures only geometries optimized from the stationary points of single amino acids in the gas phase. Indeed, PDB/NM combines the sampling of relevant dihedral angles with chemically correct local geometries. Geometries sampled using PDB/NM were used to build kriging models for alanine and lysine, and their prediction accuracy was compared to models built from geometries sampled from three other sampling approaches. Bond length variation, as opposed to variation in dihedral angles, puts pressure on prediction accuracy, potentially lowering it. Hence, the larger coverage of dihedral angles of the PDB/NM method does not deteriorate the predictive accuracy of kriging models, compared to the NM sampling around local energetic minima used so far in the development of QCTFF. © 2015 The Authors. Journal of Computational Chemistry Published by Wiley Periodicals, Inc.

## Introduction

The rapid but accurate evaluation of potential energy for biomolecular simulation continues to be a challenge. Next generation force fields, which could eventually replace the traditional force fields, continue to be developed. Among the former are AMOEBA,^1^ XED,^2^ SIBFA,^3^ and ACKS2,^4^ which all advocate multipolar electrostatics,^5^, ^6^ absent in classical architectures.^7^, ^8^ The Quantum Chemical Topological Force Field (QCTFF)^9^, ^10^ shares this approach to improved electrostatic energy prediction but, on top of this, introduces machine learning to handle electron density fluctuations in response to changes in nuclear configuration. QCTFF aims at capturing the end result of this polarization process rather than the process itself. The machine learning models that QCTFF depends on need to be properly trained with a sufficient number of configurations, but perhaps more importantly, with relevant configurations. The work presented here deals with this problem, and does so in the context of real protein structures.

Machine learning focuses on algorithms that can learn from data, in this case properties (multipole moments and energies) of topological atoms. Machine learning proposes computational methods that generate predictive models that map an output variable to a set of input variables. Models are then built through a training procedure using a set of input values with known output. QCTFF, which continues to be developed in our lab, is an innovative approach to predicting the energy of a molecular system much faster than first principle calculations can. For that purpose, QCTFF captures atomically partitioned first principle information of the system trained for. QCTFF achieves this by relying on a machine learning method called kriging,^11^, ^12^, ^13^ which is increasingly being used^14^, ^15^, ^16^, ^17^, ^18^, ^19^ in the community of force field and potential design.

Traditional force fields approximate energy through bonded and nonbonded contributions that incorporate often loosely defined atom types with their own set of experimentally or computationally obtained parameters. QCTFF operates outside this traditional framework: its architecture does not distinguish between bonded and nonbonded interactions, and atom types do not need to be defined. Instead, QCTFF focuses directly on how atoms interact, allowing for a spectrum of covalency rather than a bonded/nonbonded dichotomy. QCTFF maps atomic properties (the output variables) to molecular coordinates (a set of input variables) using kriging. Therefore, a QCTFF atom will be endowed with a number of kriging models, each describing how an atomic property changes as a function of the coordinates of the molecular system.

To build a QCTFF kriging model, example molecular geometries must be obtained to train the model. QCTFF development targets the simulation of biomolecules, in particular proteins, hence amino acids are molecules of key interest. When sampling amino acid geometries as input for kriging models, the sampled geometries must include all the conformations that one may reasonably expect to occur during the simulation of a protein. Our current paradigm for the sampling of molecular geometries is to use a NM sampling approach. To do this, a small number of stationary points on the potential energy surface of a given molecule of interest are located, and the NM at each stationary point (or local energy minimum) are calculated. Energy is then put randomly into the NM to distort the molecule, and “snapshots” are taken to obtain distorted geometries. The minimum energy conformations of all 20 naturally occurring amino acids have been reported in a comprehensive study,^20^ all obtained at the same level of theory. Kriging models built from NM sampled geometries have been used to predict successfully the atomic multipole moments of a range of molecules. These include small organics, amino acids, and hydrogen bonded dimers.^17^, ^21^, ^22^, ^23^, ^24^, ^25^, ^26^ Recently, the electronic kinetic energy of QCT atoms (see Quantum Chemical Topology section) has been successfully incorporated into kriging models for methanol, NMA, glycine and triglycine.^27^ Intra‐atomic terms such as the (electronic) kinetic energy are not explicitly incorporated in classical force fields but to gain an appreciation of chemical phenomena, such as steric hindrance, intra‐atomic terms have been proven important and therefore should be included in QCTFF.^28^ Some interesting work quantifies the steric effect, still within QCT, but in the context of experimental^29^ electron densities, conceptual DFT,^30^ and energy decomposition analysis.^31^

The only other alternative sampling approach investigated draws snapshots from a molecular dynamics simulation, which has been done^32^ for liquid water. In the current work, a third sampling method is investigated, one that is pivotal for a realistic sampling of amino acid conformations and one that incorporates experimental information (X‐ray structures).

Amino acids are typically described as consisting of two units: a back bone and a side chain. The conformational preference of the backbone unit is dictated by the secondary structure of the proteins and is well understood. The dihedral angles denoted Φ and Ψ describe the back bone using Ramachandran plots. These plots relate the values of Φ and Ψ to a particular secondary structure. Different amino acids display preferences for different regions of the Ramachandran plot, and a thorough investigation of the preferences for all 20 naturally occurring amino acids has been performed before.^33^, ^34^ The side chain of an amino acid may exist as a number of different rotamers depending on the side chain dihedrals. Extensive work has been undertaken by other groups to understand the relative populations of the different rotamers occupied by each amino acid, and this has led to a number of rotamer libraries being constructed.^35^, ^36^, ^37^, ^38^, ^39^, ^40^ A rotamer library is a comprehensive guide, drawn from molecular dynamics simulation or protein crystallography, detailing the statistical populations and frequencies of the dihedral angles adopted by amino acid side chains. These libraries may then be used to predict, build, design and solve new protein structures.^41^ Torsional energy terms are so important that they receive special attention in force field design, see Ref. 42 for a recent example.

Normal modes sampling has proved successful at sampling conformational space around an input energetic minimum or stationary point. However, one must consider whether the gas phase minimum energy geometries of an amino acid accurately mimic the amino acid structures found in proteins. We note that, in more general terms, the biases induced by datasets that are restricted to stationary or only little deformed structures were also discussed within the context of DFT.^43^ It is accepted that amino acids and polypeptides have an intrinsic propensity for specific molecular configurations, and that this preference can differ depending on whether the amino acid exists in a folded protein tertiary structure or a disordered, solvated state.^44^ Ramos and coworkers^45^ performed *ab initio* calculations on all 20 natural amino acids using both gas phase and PCM solvation. Of the 323 chemical bonds and 469 angles present, they found mean unsigned errors of less than 0.02 Å and 3° between the PCM and gas phase bonds and angles, respectively. However, the environment of a globular protein is different to that of a hydrated polypeptide due to a number of factors such as intraresidue hydrogen bonding and steric considerations that have an effect on the amino acid conformation.

The work of Jha et al.^46^ clearly shows the effect of the environment on the backbone angles Φ and Ψ. They compared the geometric preferences of all 20 amino acids using data from two protein coil libraries: one including residues in structural motifs, and the other only those residues in disordered sections of the proteins. The ratios of structures found in the β‐sheet, PPII and α‐helical regions were clearly different between the two libraries. To further demonstrate the effect of environment on the structural preferences of amino acids, the distribution of structures obtained from both coil libraries also differed significantly from those obtained experimentally for the central residue of Gly‐X‐Gly tripeptides (where X is a naturally occurring amino acid).^47^, ^48^ It has been shown, both experimentally (using NMR J couplings) and computationally, that disordered amino acid residues favor specific regions of the Ramachandran plot (typically β‐sheet and PPII regions) in contrast to the conformational populations found in ordered protein secondary structures.^44^, ^46^, ^49^, ^50^, ^51^ It has also been shown that the side chain rotamer preference of an amino acid is related to the secondary structure of the polypeptide in which it resides,^52^ and this relationship between environment and structure has been used successfully in rotamer libraries to predict side chain conformations.^53^ In the long term, these results imply that gas phase energy minima of single amino acids used to sample geometries from, are insufficient to sample all important chemically relevant structures.

The efficient sampling of molecular geometries is a challenging problem due to the rapid increase in the available conformational space as molecules grow in size. A systematic search of conformational space to find low energy structures is impractical and inefficient. A number of efficient approaches have been presented in the literature including the use of molecular dynamics,^54^, ^55^ Monte Carlo,^56^ transition path sampling,^57^, ^58^, ^59^ and metadynamics.^60^ Additionally, fragment based approaches may be used to improve a systematic approach by reducing the number of conformations searched though elimination processes. An example of such an approach is that of Luo and coworkers^61^ where, by fragmenting the Gly‐Tyr‐Gly‐Arg tetrapeptide, they reduced 19.6 billion possible candidates for the global minimum conformation down to only 5760.

An alternative to computational sampling approaches for finding important amino acid geometries is to source them from protein crystal structures. Unfortunately, crystal structures cannot be used directly as input into kriging models for several reasons. First, only heavy atoms are detectable by X‐ray crystallography and so the hydrogen atom coordinates are dependent upon the refinement process used. Second, removing an amino acid from a crystal structure breaks the peptide bonds at either end of the backbone, which drastically changes the chemical environment and results in incomplete valence of the terminal atoms. Therefore, some post‐Protein Databank (PDB)‐extraction modifications to the sampled amino acids are required before input to QCTFF. Thirdly and finally, the resolution of the atomic coordinates varies from one crystal structure to another, and sometimes unrealistic bond lengths and angles may be present within a crystal structure. To address the above concerns, a novel sampling approach is presented here. This approach samples amino acids from the PDB, relaxes bond lengths, and valence angles by an *ab initio* method while preserving the dihedral angles, and then performs nonstationary NM sampling around each sampled amino acid. This approach is termed PDB/NM and the details of both sampling approaches are explained in the following sections.

## Background and Methods

Because many of the technical points concerning QCTFF have been described in detail in previous work of our lab, we only give a brief overview of the key concepts here. A comprehensive introduction to kriging and how it features in QCTFF is given in Ref. 19 while Refs. [24,25] provides the most up‐to‐date detail on the overall training procedure of QCTFF, now called GAIA. Additional descriptions of the machine learning method are also provided in Refs. [17,26].

### Quantum chemical topology

Underpinning the development of QCTFF^9^ is Quantum Chemical Topology (QCT),^62^ which embraces all work^63^ in quantum chemistry that uses the topological language of dynamical systems (e.g. attractor, basin, homeomorphism, gradient path, separatrix, critical points). QCT contains the “quantum theory of atoms in molecules”^64^, ^65^, ^66^ as a special case where this topological language is applied to the electron density ρ and its Laplacian. A topological atom Ω_A_ is a bundle of gradient paths (i.e., trajectories of steepest ascent through ρ), terminating at a maximum critical point, which typically coincides with the nucleus A. Topological atoms are defined in a parameter‐free manner, and they are nonoverlapping and sharply bounded (at the inside of the molecule) by so‐called interatomic surfaces.

It is a good idea to expand the 1/*r*_12_ expression occurring in the equation for the Coulomb energy between two electron densities. A popular and compact expansion introduces spherical harmonics, which in turn lead to atomic multipole moments. Multipole moments are able to describe the anisotropy^67^ of the electron density, in contrast to (isotropic) point charges used by popular force fields such as AMBER^68^ and CHARMM.^69^ The charge of an atom is the zero‐order term of the multipolar expansion, and it is only by including higher‐order terms that the anisotropy of the electron density is described. There is considerable evidence, as collected in a recent review,^5^ of the advantages of multipolar electrostatics over point charges. QCTFF incorporates multipolar electrostatics, and in the current work it is the atomic multipole moments that are the topological property of interest, that is, they are the output that kriging is tasked to predict.

The Coulomb interaction between two topological atoms $\Omega_{A}$ and $\Omega_{B}$ is given^70^ by

(1) $$E_{AB}^{Coul}=\sum_{l_{A}l_{B}m_{A}m_{B}}Q_{l_{A}m_{A}}T_{l_{A}l_{B}m_{A}m_{B}}Q_{l_{B}m_{B}}$$

where $Q_{l_{A}m_{A}}$ is a multipole moment and $T_{l_{A}l_{B}m_{A}m_{B}}$ is the interaction tensor between two multipole moments. A convenient concept when dealing with the electrostatic interaction between two multipole moments of order $l_{A}$ and $l_{B}$ is the interaction rank, L, given by:

(2) $$L=l_{A}+l_{B}+1$$

It has been shown that interaction rank L=5 provides a satisfactory description of the electrostatics acting in system.^71^, ^72^ Note that L=5 requires all atomic multipole moments up to and including hexadecupole (fourth order multipole moments, ℓ = 4) to be calculated, resulting in 25 multipole moments for each atom.

Atomic properties other than multipole moments may be obtained from QCT. The interacting quantum atoms (IQA)^73^ method is a well‐developed topological energy decomposition scheme based on the calculation^74^ of the exact nonexpanded topological Coulomb energy. IQA decomposes a molecular system in a combination of both intra‐atomic (“self”) and interatomic energy terms. Details of the decomposition scheme are beyond the scope of this article but QCTFF is currently incorporating the non‐Coulomb terms by the same kriging treatment as the atomic multipole moments in the current work.

### The atomic local frame and kriging

QCTFF uses kriging,^11^, ^13^, ^75^ also known as Gaussian process regression,^12^ which is a method of capturing the changes in atomic multipole moments as a function of molecular geometry. A detailed description is provided in earlier work^25^ so only a brief description is provided here. As the coordinates of an atomic system evolve, for example when bonds stretch and angles bend, the topological properties of the atoms involved will change, e.g. example their atomic charges (or monopole moments). Using kriging, it is possible to build models capable of predicting changes in an atomic property by evaluating the molecular coordinates. In the present work, kriging models are built for the first 25 atomic multipole moments (up to, and including, hexadecapole moment) of each atom in the amino acids alanine (Ala) and lysine (Lys). By treating the atomic multipole moments in this way, both polarization and charge transfer effects are captured.

A chemical system may be defined by a minimum of 3*N*−6 internal coordinates. In the language of machine learning, the 3*N*−6 coordinates around an atom are referred to as features, and it is these features that a multipole moment is mapped to. In QCTFF an atomic local frame (ALF) is defined to describe the 3*N*−6 coordinates around a central atom. Consider a central atom, denoted A. First, the Cahn–Ingold–Prelog rules are used to determine the two atoms of highest priority bonded to A, and these atoms are termed X and Y in order of priority. The distances $R_{AX}$ and $R_{AY}$, and the angle $\theta_{XAY}$ define the three ALF coordinates. Subsequently a right‐handed coordinate system is stabilized using the *XAY* plane. All other atoms in the system can then be described by three polar coordinates, $R_{AK},\phi_{AK}$, and $\theta_{AK}$. One therefore obtains *N*−3 sets of three spherical polar coordinates each, which combined with the aforementioned ALF coordinates make up the 3*N*−6 coordinates required, that is, 3(*N*−3)+3 = 3*N*−6.

Returning to kriging, the change in a given multipole moment is smooth with respect to a change in the ALF coordinates. Therefore it is safe to interpolate the atomic multipole moments of an unknown molecular geometry existing inside a set of known geometries. Kriging is used to build models capable of accurate interpolation of the atomic multipole moments by mapping an input (nuclear coordinates) to an output (a multipole moment). To achieve this, a training set of molecular geometries with known atomic multipole moments is required. The sampling of molecular geometries for training kriging models is described below. Kriging models calculate atomic multipole moments of a new geometry by the following process:

(3) $$\hat{y}(x*)=\hat{\mu }+\sum_{i=1}^{n}a_{i}\cdot r_{i}$$

where $\hat{y}(x*)$ is a multipole moment at a new set of coordinates x* and $\hat{\mu }$ is the global (average) value of the moment. $The\;factor\;a_{i}$ is the ith element of the vector $a=R^{‐1}\left(y-1\hat{\mu }\right)$ and $r_{i}$ is the ith element of r, defined by

(4) $$r={\left\{\text{cor}[\varepsilon (x*),\varepsilon (x^{1})],cor[\varepsilon (x*),\varepsilon (x^{2})],...,\text{cor}[\varepsilon (x*),\varepsilon (x^{n})]\right\}}^{T}$$

where T marks the transpose.

Kriging treats all moments as an error from the global value, and it is the correlation of these errors for a given multipole moment between all n training points that is calculated by kriging. This is achieved by building a n×n correlation matrix R between all pairs of training points with elements *R_ij,_* given by

(5) $$R_{ij}=\text{cor}[\varepsilon (x^{i}),\varepsilon (x^{j})]=\exp\left[-\sum_{h=1}^{d}\theta_{h}|x_{h}^{i}-x_{h}^{j}{|}^{p_{h}}\right]$$

where $x^{i}$ and $x^{j}$ are training points composed of d features. The parameters $\theta_{h}$ ( $\theta_{h}\geq 0$) and $p_{h}$ ( ${1<p}_{h}\leq 2$) describe the importance of each feature h and may be written as the *d*‐dimensional vectors θ and p. A large value of $\theta_{h}$ corresponds to a feature being highly correlated to the output multipole moment. The parameter $p_{h}$ describes the smoothness of the function, and is often close to 2.

A second crucial concept underpinning kriging is the so‐called concentrated (or reduced) log‐likelihood function $\hat{L}$, defined as

(6) $$\hat{L}(\theta ,p)=-\frac{n}{2}\log({\hat{\sigma }}^{2})-\frac{1}{2}\log(|R|)$$

where

(7) $${\hat{\sigma }}^{2}=\frac{{\left(y-1\hat{\mu }\right)}^{T}R^{-1}(y-1\hat{\mu })}{n}$$

and

(8) $$\hat{\mu }=\frac{1^{T}R^{-1}y}{1^{T}R^{-1}1}$$

where y is a vector of response values for each training point and **1** is a vector of 1s. Another (very different) machine learning method called particle swarm optimization^76^ then searches for the optimum values of θ and p that maximize the concentrated log‐likelihood function.

In Quantum Chemical Topology section, it was stated that each atom is described by 25 multipole moments, and therefore there are 25 kriging models associated with each atom. The kriging models are tested on an external test set of geometries, which is strictly not part of the training set. For each test molecule, we predict all the multipole moments of all the atoms in the system, and then calculate all electrostatic interactions between atoms separated by a minimum of three covalent bonds (i.e., 1, *n* and *n* > 3 interactions). Each predicted interaction energy (between two atoms A and B) is then compared to the original (i.e., not trained) interaction energy obtained from the original (i.e., not kriged) atomic multipole moments. Then the errors of all the aforementioned interactions within one molecular geometry are summed. The absolute value of this summed error (for each test geometry) will be plotted against percentile (i.e., % of test geometries) to obtain a called S‐curve. Each point on such a curve corresponds to this final absolute error (i.e., |Δ*E*_system_|) in eq. (9)). The S‐curve will be described later when one is obtained. The complete description of errors just mentioned is expressed is eq. (9),

$$\left|\Delta E_{system}\right|=\left|E_{system}^{original}-E_{system}^{predicted}\right|=|\sum_{AB}E_{AB}^{original}-\sum_{AB}E_{AB}^{predicted}|$$

(9) $$=|\sum_{AB}\left(E_{AB}^{original}-E_{AB}^{predicted}\right)|$$

### PDB sampling method

**Figure 1:**
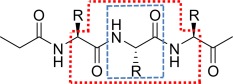
Diagrammatic representation of the atoms extracted by MOROS including the target amino acid (blue box) and also the full set of atoms including those used to make the peptide caps (red box).

PDB sampling is performed by the in‐house (scripting) code MOROS and is used to extract all seed geometries of a particular amino acid from a set of crystal structures. A list of the 260 PDB crystal structure codes sampled from is provided in Part A of the Supporting Information. Hydrogen atoms were added to all protein crystal structures using the HAAD code of Li et al.^77^ The HAAD algorithm was developed to add accurately hydrogen atoms by analyzing the positions of nearby heavy atoms, following the basic rules of orbital hybridization and through optimization of steric and electrostatic parameters. HAAD was found to outperform the popular software CHARMM and REDUCE^78^ with the RMSD of predicted hydrogen atom positions decreased by 26% and 11%, respectively, when compared to high resolution X‐ray and neutron diffraction structures. MOROS returns as output “capped” amino acids meaning that H_3_CC(=O)— and —N(H)CH_3_ are appended at the N and C termini of the sampled amino acid, respectively. These atoms are included so that the peptide bonds remain intact, and thereby yield a more realistic representation of an amino acid while present in a protein. The capping groups are built by extracting the atomic coordinates from the residues preceding and following the residue of interest. Figure 1 shows the atoms extracted by MOROS including the amino acid of interest (blue box), and also atoms that make up the caps (red box).

In preparation for nonstationary NM treatment, the sampled amino acid geometries are then allowed to partially geometry‐relax, that is under the restriction of fixed dihedral angles. This stage is important as it removes some of the outlying bond lengths originally present due to the poor quality crystal structure resolution.

The next step in PDB sampling is to perform a frequency calculation on each amino acid geometry, by first obtaining the Hessian of the potential energy on that point of the surface, for input for the non‐stationary NM sampling of the geometry. A choice must be made regarding the number of PDB‐sampled amino acid geometries to use as input for nonstationary NM, as this choice influences the number of geometries sampled using NM. This choice is investigated in Results and Discussion section, and unless otherwise stated, 300 random PDB‐sampled amino acid geometries are input to the nonstationary NM. The combined PDB and nonstationary NM sampling method will henceforth be referred to as PDB/NM.

## “Normal Modes” sampling

Typical normal mode analysis is conducted at an energetic minimum (or stationary point) on the molecular potential energy surface. However, the mathematics leading to NM does not restrict their use only at stationary points. A simple generalization of the derivation of the molecular NM enables their evaluation at nonstationary points on the potential energy surface. This derivation is provided in Part B of the Supporting Information. In the following, we present a conformational sampling methodology, which uses these “non‐stationary point normal modes” as a means for distorting a molecule, that is, sample its configurations. By diagonalization of the mass‐weighted Hessian, **H**, the frequency of each of the *N*_vib_ = 3*N* – 6 NM is evaluated. These *N*_vib_ NM are orthogonal and form a complete basis within which internal molecular motions can be described. With the mass‐weighted force vector, **F**, a set of *N*_vib_ harmonic equations of motion is obtained. These equations of motion allow us to distort the molecular geometries, and perform a sampling of conformational space.

We now discuss the computational means utilized to obtain the various parameters required to evolve the NM. This subsequently permits us to obtain a set of geometries we consider representative of realistic vibrational states of a molecular system. What follows is a brief paraphrase of the excellent explanation given by Ochterski.^79^ Beginning with the transformation from the mass‐weighted Cartesian coordinates, q, to the set of $N_{vib}$ internal coordinates, s, we construct the 3N×3N transformation matrix, D, satisfying

(10) s=Dq 

Outlining the construction of D is beyond the scope of this article. Suffice to say that six orthonormal vectors occupy the first six columns of D, and correspond to the global translational and rotational motions of the system (as given by the Sayvetz conditions). The remaining *N*_vib_ vectors are generated by means of a Gram–Schmidt orthonormalization procedure.

The mass‐weighted force F and the mass‐weighted Hessian H, both outlined in Part B of the Supporting Information, are transformed into the internal coordinate basis, by use of D

(11) $$F_{s}=D\;F_{q}H_{s}=D^{⊤}H_{q}D$$

where the subscripts denote the basis in which these quantities are expressed and ^T^ denotes the transpose. To evaluate the frequencies of the various modes of motion, we diagonalize $H_{s}$,

(12) $$E^{‐1}H_{s}E=I\lambda$$

where E denote the eigenvectors of $H_{s}$ and I is the identity matrix. The resultant eigenvalues, ${{\left(I\lambda \right)}_{ii}=\lambda }_{i}$, are related to the mode frequencies, $\nu_{i}$, by

(13) $$\nu_{i}=\sqrt{\frac{\lambda_{i}}{4\pi^{2}c^{2}}}\;\forall i=1,\ldots ,3N$$

where c is a factor comprising the speed of light and the conversion between atomic units and cm^−1^. Of course, six of these frequencies correspond to the global translational and rotational degrees of freedom of the system, thus yielding *N*_vib_ nonzero frequencies. The reduced masses and force constants, corresponding to the modes with nonvanishing frequency, are given by similar manipulations of these quantities. The reader is again directed to Ochterski^79^ for a discussion of their calculation.

The amplitude of the ith mode, $A_{i}$, is given by rearrangement of the familiar expression for the energy of a simple harmonic oscillator

(14) $$A_{i}=\sqrt{\frac{2E}{k_{i}}}$$

where $k_{i}$ is the force constant of the mode of motion, and E is the energy available to it. We now have all quantities required to evolve the modes of motion and replicate the vibrational dynamics of the system. The total energy available to the system is given by the expression for thermal energy, $E=N_{vib}kT/2$, and is stochastically distributed throughout the modes. A temperature of 298 K was used throughout this work. The phase factors of the modes, ϕ, are also randomly assigned: if ϕ=0 for all modes, then they oscillate in unison, which is physically unrealistic. Instead, we assume the modes to resonate out of phase with one another, as energy transfer to each mode from an external heat bath will be strongly decoherent.

Let us note that the average thermal energy available to each mode will comply with a standard equipartition of energy for a physically realistic sampling methodology. The energy available to each mode is then subjected to small stochastic fluctuations. However, one deduces from the above description of our own methodology that we did not follow the route of equipartition. The driving force for this decision was to increase the domain of conformational space, which is then accessible to our sampling methodology. As explained above, we have chosen to distribute the total thermal energy stochastically through all modes. Given a standard equipartition of thermal energy, the *i*th mode, *q_i_*, is limited to the domain *q_i_* ^0^ *− A_i_/2 ≤ q_i_ ≤ q_i_* ^0^ *+ A_i_/2*, where *q_i_* ^0^ is the reference state of the mode and *A_i_* is given in eq. (14). However, by stochastically distributing the thermal energy through the modes, the energy available to the *i*th mode, *E_i_*, can then take any value in the range 0 *≤ E_i_ ≤ nk*_B_ *T/2,* as long as the sum of the *E_i_* is *nk*_B_ *T/2*. In this sense the currently applied methodology is more general than that of the equipartition. If *E_i_* takes the value of *k*_B_ *T/2* for all modes, then the sampling domain coincides with the sampling domain of a standard equipartition of energy. However, all other combinations of the *E_i_* have different sampling domains. The sampling domain that is accessible to our stochastic distribution of thermal energy through the modes is then the union of all sampling domains that arise from all possible combinations of the *E_i_*. We therefore obtain the largest sampling domain possible for our methodology, which is necessary for the construction of a widely applicable kriging model.

Two issues arise with stochastically distributing the thermal energy through the modes, one methodological and one conceptual. The methodological concern is that there is a non‐negligible probability for a significant proportion of the available thermal energy being placed into one mode. If this mode is strongly linked to the motion of a bond length or valence angle, then there is the potential for sampling nonphysical geometries. We have implemented a filtering procedure that prevents the output of such nonphysical geometries. Consider a bond between atoms A and B, of length ℓ_AB_, within a seed geometry. If ℓ_AB_ exceeds a value of *k*_BOND_ multiplied by the sum of the atomic covalent radii, (*r*_A_ *+ r*_B_), then the geometry is considered nonphysical and rejected. Similarly, if ℓ_AB_ is lower than the inverse of *k*_BOND_ multiplied by the sum of the atomic covalent radii, the bond is considered too short and rejected. In other words, every bond length must obey the inequality (*1/k*_BOND_)(*r*_A_ *+ r*_B_ *) ≤* ℓ_AB_ *≤ k*_BOND_(*r*_A_ *+ r*_B_). Valence angles undergo a similar treatment, so that given any valence angle of the seed geometry, α_0_, the corresponding valence angle of the sampled geometry, α, must obey the inequality α_0_ */k*_ANGLE_ *≤* α *≤ k*_ANGLE_α_0_. In the following work, the “stretching” parameters, *k*_BOND_ and *k*_ANGLE_, were both set to 1.20. The conceptual concern that we mentioned is that distributing the thermal energy stochastically throughout the modes is nonphysical in terms of equilibrium thermodynamics. For our purposes we are more interested in sufficiently large sampling domain.

The sole remaining issue is the choice of a dynamical time step with which to evolve the various modes of motion. We ensure that a single oscillation of a mode is sampled uniformly. In other words, for a complete cycle of the *i*th harmonic equation of motion, the time period of the mode is $T_{i}=1/\nu_{i}$. A parameter, $n_{cycle}$, defines the number points to be evaluated along a single cycle of the harmonic equation of motion. From this, we define the quantity $\Delta t_{i}=T_{i}/n_{cycle}$, which is the dynamical timestep for the equation of motion. The quantity $n_{cycle}$ is left as a user‐defined input, and is set to $n_{cycle\;}$= 10 from now on. Additionally, the distribution of the total energy throughout the modes is considered a dynamic quantity, and so for every $n_{reset}$ samples that are output, the energy is randomly redistributed throughout the system. The phase factors are also redefined at the same frequency. Again, $n_{reset}$ is left as a user‐defined parameter, and is set as $n_{reset}=$ 2 in the following. A further justification for the way we sample is given in Part C of the Supporting Information.

### Computational details

**Figure 2:**
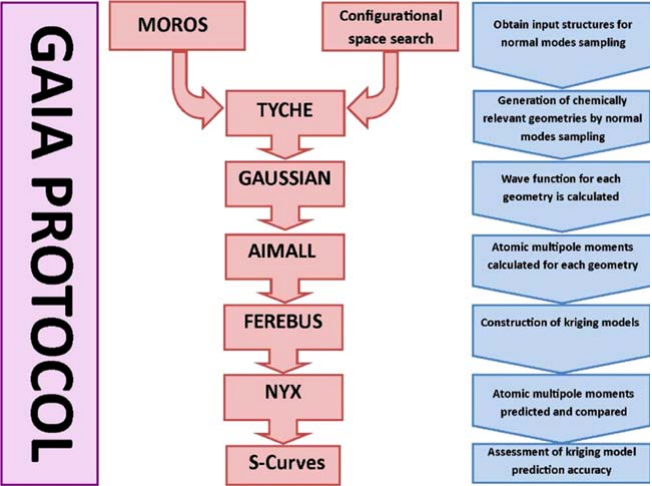
The fully automated GAIA protocol followed to obtain and to test kriging models.

Sampling of amino acids from the crystal structures was performed by the in‐house code MOROS while the in‐house FORTRAN code TYCHE distorted the geometries according to NM. The fully automated GAIA code (formerly named AUTOLINE in previous work) was used to build the training and test sets of molecular geometries (Fig. 2). An expanded flow chart of the GAIA procedure is given by Fletcher et al.^24^ Once the sampled amino acid geometries were obtained from either PDB/NM or NM, the molecular wave function for each geometry was obtained at the B3LYP/aug‐cc‐pVDZ level using GAUSSIAN09.^80^ The FORTRAN program AIMAll^81^ obtained the atomic multipole moments. The parameters briaq = auto and boaq = high, which are standard in GAIA, because boaq = high has been seen in the past as a good compromise between accuracy and speed. Kriging models were built and then tested using the in‐house codes FEREBUS and NYX, respectively. All kriging models were built using *N*_train_ = 1000 training geometries and were tested on 400 randomly selected geometries from the remaining 1000. Experience has shown that kriging models deteriorate in prediction quality as the standard integration error (i.e., the familiar Lagrangian *L* of atom Ω or *L*(Ω)) increases. Hence it is best to set *L*(Ω) as low as possible but this norm causes an increasing number of integrations to have to be discarded. A good compromise is allowing a maximum integration error of *L*(Ω)=0.001 a.u. This value was enforced throughout this work, which keeps the number of discarded atoms reasonable but not nil, explaining the surplus of sampled geometries at the outset.

## Results and Discussion

Kriging models were built for the two amino acids alanine (Ala) and lysine (Lys) using geometries sampled from four different sampling approaches: PDB_NO_OPT, PDB_OPT, NM and PDB/NM. These four methods are described in Table 1.

**Table 1:** An overview of the four sampling approaches.

PDB_OPT	Molecular geometries sampled directly from crystal structure coordinates and H atoms added by the HAAD program. GAUSSIAN fully optimizes bond lengths and valence angles but all dihedral angles remain fixed.
PDB_NO_OPT	Molecular geometries taken directly from PDB coordinates and H atoms added by HAAD. Single‐point GAUSSIAN calculations without any geometry relaxation.
NM	Standard NM sampling procedure using TYCHE to sample molecular geometries from a number of local energy minima in the gas phase. The local energy minima themselves are not included in either training or test sets.
PDB/NM	300 randomly selected PDB “seed geometries” sampled with PDB_OPT, each acquiring 7 geometries generated from the nonstationary NM. The “seed geometries” themselves are not included in either training or test sets.

**Figure 3:**
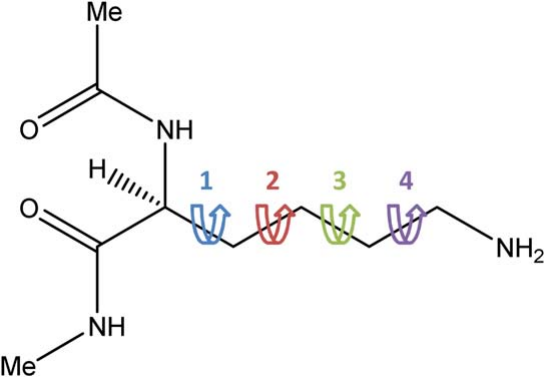
The four dihedral angles in the side chain of Lys, referred to as *χ*_1_ (blue), *χ*_2_ (red), *χ*_3_ (green), and *χ*_4_ (purple). [Color figure can be viewed in the online issue, which is available at wileyonlinelibrary.com.]

Alanine was chosen because it is the smallest amino acid with a (nontrivial) side chain. Because there is only one side chain dihedral angle (*χ*_1_), as opposed to the four dihedral angles (*χ*_1_, *χ*_2_, *χ*_3_, *χ*_4_) controlling the side chain of lysine, the ϕ and ψ angles dominate the dihedral motion of alanine. Lysine has the most flexible side chain of all 20 naturally occurring amino acids, and therefore has been chosen as a rigorous test of the performance of kriging when dealing with highly flexible molecules. Figure 3 shows the four side chain dihedrals in lysine around C—C bonds or *χ*_1_, *χ*_2_, *χ*_3,_ and *χ*_4_.

### Testing the PDB/NM sampling approach

**Figure 4:**
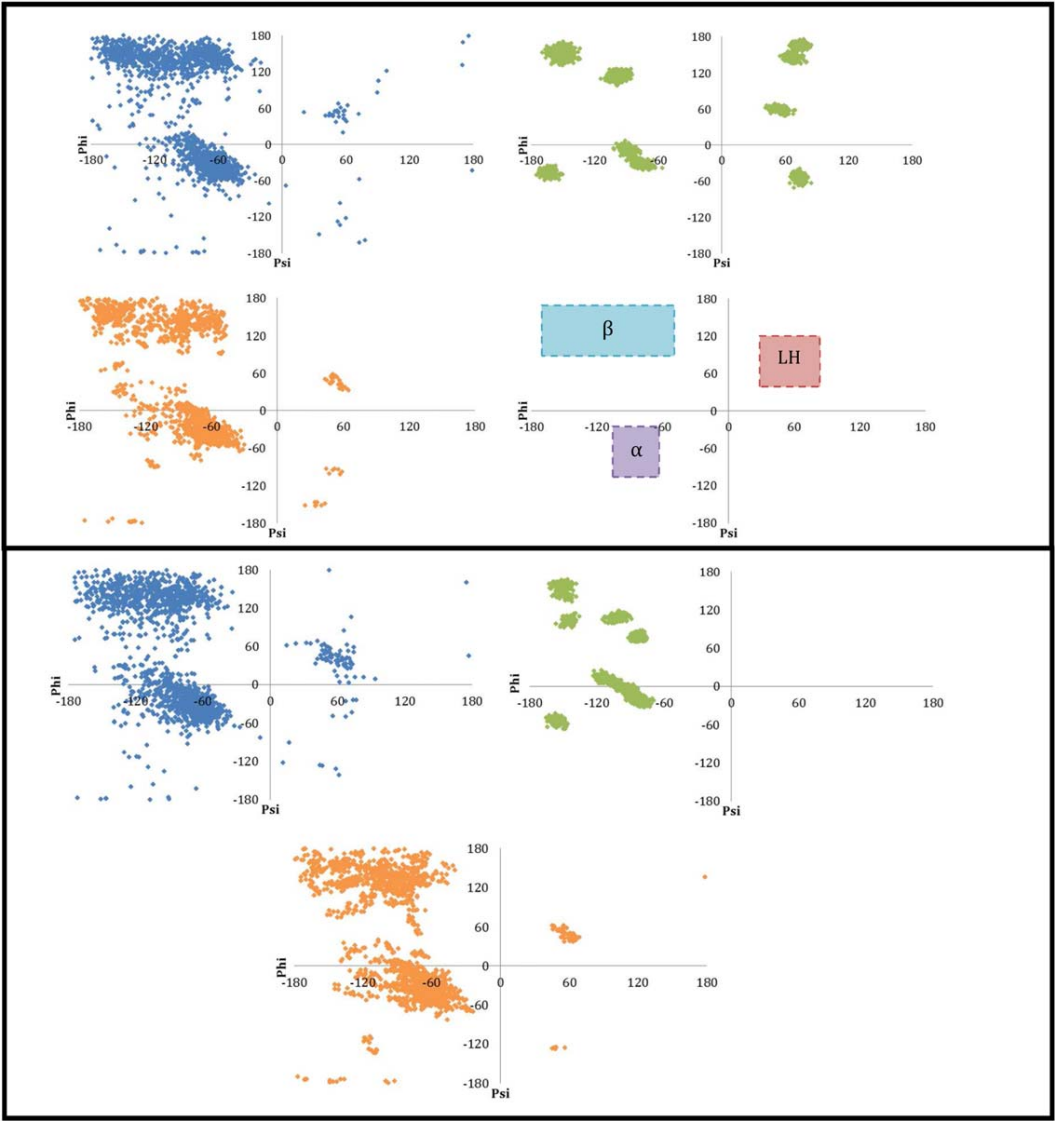
Ramachandran plots of Ala (top box) and Lys (bottom box) sampled using PDB_OPT and PDB_NO_OPT (blue), NM (green), and PDB/NM (orange). In the bottom right panel of the top box is a guide to the regions corresponding to the secondary structural motifs, β‐sheet (labeled β), α‐helix (labeled α), and left‐handed alpha helix (labeled LH). [Color figure can be viewed in the online issue, which is available at wileyonlinelibrary.com.]

Kriging models were built for the amino acids Ala and Lys using the four sampling strategies defined in Table 1. Ramachandran plots for the sampled alanine geometries by each of the sampling methods are shown in Figure 4. The dihedral angles are fixed to the same values in both the PDB_OPT and PDB_NO_OPT approach, which is why Figure 4 assigns the same color (blue) to the distribution of ψ and ϕ angles of their geometries. As expected, the PDB‐sampled Ramachandran plots for both Ala and Lys display a sampling bias toward the α‐helix and β‐sheet regions with additional clusters of geometries in the left‐handed helix region. The green Ramachandran plots display the sampled geometries obtained by the NM method. A number of islands of geometries around the gas‐phase energy minima are observed. Several islands are clearly disconnected but some may overlap, such as the long island in lysine (bottom box) at the bottom right of the whole cluster of islands. Because there are regions of conformational space populated by the PDB sampling approaches but not the NM approach, we conclude here that NM sampling from gas phase energy minima is inadequate for building kriging models to be used in biomolecular simulation. This is most noticeable in the case of Lys, where the NM Ramachandran plot appears sparsely populated compared to both the other sampling methods and the Ala NM Ramachandran plot. This is because the side chain of lysine is very flexible, and for each of the nine actual islands in the Ramachandran plot, there are multiple overlapping energy minima with different side chain conformations. This explains why the 39 input minima only appear as nine islands on the Ramachandran. The orange Ramachandran plots, containing the Ala and Lys geometries sampled by the PDB/NM approach, strongly resemble the plots of both PDB_OPT (blue) and PDB_NO_OPT (blue) but with fewer points in regions away from the α‐helix and β‐sheet region. This is because the 300 “seed” geometries used as input for the NM sampling were randomly selected from the PDB_OPT sampled geometries and, statistically, they are most likely to be sampled from these well populated α‐helix and β‐sheet regions. The benefit of PDB/NM (orange) is that, on top of realistic distributions of dihedral angles, bond lengths and angles are more realistic and they are both varied.

**Figure 5:**
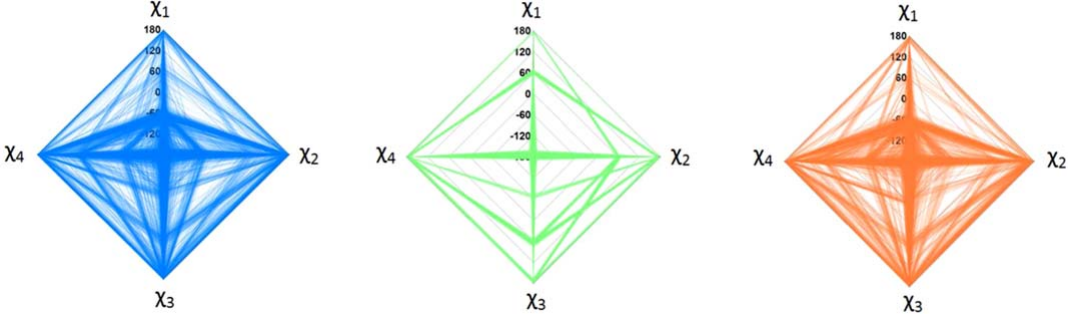
Spider plots displaying the Lys side chain conformations sampled by each of the four sampling approaches: PDB_OPT and PDB_NO_OPT (blue), NM (green), and PDB/NM (orange). Each axis ranges from −180^°^ to 180^°^. [Color figure can be viewed in the online issue, which is available at wileyonlinelibrary.com.]

Figure 5 shows so‐called spider plots of the side chain dihedral angles sampled by each of the sampling approaches. In a spider plot, each of the four axes (meeting at the origin) corresponds to all values that each of the four side chain dihedrals *χ_n_* (*n* = 1, 2, 3, or 4) can adopt, that is, from −180° to 180°. Each sampled geometry then corresponds to a quadruplet of dihedral values (*χ*_1_, *χ*_2_, *χ*_3_, *χ*_4_), each marked by a point on each of the four corresponding axes. These four points are then linked by four colored lines, which form a (typically lozenge‐like) pattern. From the density of these patterns one obtains an instant glimpse of the conformational diversity (or lack thereof) of the side chain geometries.

Clearly, the NM sampling approach (green) samples a very limited range of side chain geometries and does not return the regions of high sampling frequency obtained by the PDB_OPT and PDB_NO_OPT (blue) approaches. For example, the gauche*^−^* (−60°) conformation of *χ*_1_ is the most sampled conformation in the protein crystal structures but this conformation is not at all present in NM. The preference of *χ*_1_ to be in the gauche^−^ conformation in proteins is a well‐documented phenomenon^35^ and thus NM sampling's shortcomings are highlighted. The PDB/NM spider plot (orange) shows a better sampling of side chain dihedral angles than that of NM. However, the former shows a sparser sampling of the less populated combinations of dihedral angles compared to PDB_OPT and PDB_NO_OPT (blue).

Table 2 presents a summary of the relative performance of each sampling approach and the resulting kriging model accuracy for both amino acids. The range in the B3LYP/aug‐cc‐pVDZ energy of the Ala and Lys geometries sampled by each of the four methods is also included in Table 2. For both amino acids the NM sampled geometries show the smallest range in *ab initio* energy. This is because the NM sampling method uses the lowest energy gas phase conformations as the input minima, and hence all sampled geometries from this method are distortions of these low energy geometries. Therefore, large deviations from the various energy minima cannot occur because the distorted geometries are confined by their respective well. This situation is different to that found in PDB geometries. Here, the lysine geometries sampled by the PDB/NM method have the largest range in *ab initio* energy, 421 kJ mol^−1^, which is much larger than found in any other sampling approach. This is expected as the PDB/NM geometries undergo substantial dihedral sampling, as well as bond length and angle distortions caused by the nonstationary NM sampling.

**Table 2:** Statistical information detailing the sampling of Ala and Lys by the four sampling methods.

	PDB_OPT	PDB_NO_OPT	NM	PDB/NM
Alanine				
Range in *ab initio* Energy	132.5	281.0	84.4	111.0
Average Bond Length Range^a^	0.02	0.07	0.11	0.12
C_α_—C_β_ Bond Length Range	0.03	0.22	0.14	0.14
Average $\left|\Delta E_{\text{system}}\right|$ b	0.7	1.8	4.0	3.4
Average $\left|E_{\text{AB}}^{\text{original}}-E_{\text{AB}}^{\text{predicted}}\right|$	0.1	0.2	0.4	0.4
Max $\left|\Delta E_{\text{system}}\right|$	6.8	25.8	18.4	17.2
Max $\left|E_{\text{AB}}^{\text{original}}-E_{\text{AB}}^{\text{predicted}}\right|$	10.0	9.4	13.7	9.4
Lysine				
Range in *ab initio* Energy	126.0	310.6	111.1	420.9
Average Bond Length Range^a^	0.02	0.08	0.13	0.14
C_α_—C_β_ Bond Length Range	0.05	0.12	0.13	0.13
Average $\left|\Delta E_{\text{system}}\right|$	1.6	2.5	3.3	3.8
Average $\left|E_{\text{AB}}^{\text{original}}-E_{\text{AB}}^{\text{predicted}}\right|$	0.2	0.3	0.3	0.4
Max $\left|\Delta E_{\text{system}}\right|$	20.4	23.1	15.2	18.1
Max $\left|E_{\text{AB}}^{\text{original}}-E_{\text{AB}}^{\text{predicted}}\right|$	32.5	34.2	7.1	28.4

All energies are in kJ mol^−1^ and all distances in Å.

a The set of training geometries provides a range (i.e., maximum–minimum) for each bond length. The ranges of all bonds appearing in the system are then averaged (over these bonds).

b The symbols referring to all energetic quantities (except the range) in this table also appear in eq. (9).

Table 2 also lists the average bond length range for all bonded atom pairs in the sampled Ala and Lys geometries, calculated for each sampling method. For both Ala and Lys, PDB_OPT yields the lowest average bond length range, 0.02 Å, due to the relaxation of the bonds to their optimal lengths (and obviously no bond length variation is introduced by NM). The average bond length ranges of 0.07 Å and 0.08 Å for PDB_NO_OPT Ala and Lys, respectively, are the next lowest values. The reason for the low average bond length range of the PDB_NO_OPT geometries is that the hydrogen addition software used, HAAD, add hydrogens at a fixed length of 0.985 Å. Therefore, the average range in bond length is reduced by all bonds containing a hydrogen atom. A more informative metric to describe the sampling of bond lengths by each method is to study the range of a single bond containing two heavy atoms. The bond between C_α_ and C_β_ was chosen for this purpose. Again, the PDB_OPT showed the lowest ranges of 0.03 and 0.05 Å, respectively, but the PDB_NO_OPT Ala geometries showed the highest range in C_α_—C_β_ distance of 0.22 Å as expected. NM and PDB/NM showed the same range in C_α_—C_β_ bond length of 0.14 Å. This highlights the similarity of both the stationary and nonstationary NM sampling algorithms in TYCHE.

**Figure 6:**
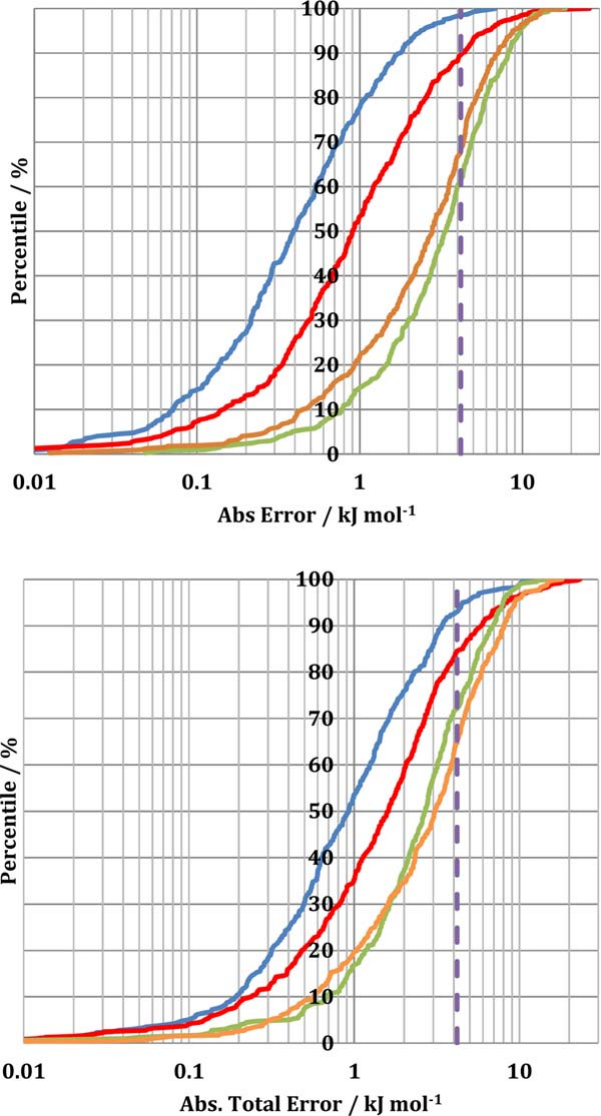
Errors in the predicted total electrostatic interaction energies (1–4 and higher) of alanine (top) and lysine (bottom) for kriging models trained with molecular geometries obtained by: PDB_OPT (blue), PDB_NO_OPT (red), NM (green), and PDB/NM (orange). The dashed purple lines mark the 1 kcal mol^−1^ threshold. [Color figure can be viewed in the online issue, which is available at wileyonlinelibrary.com.]

Kriging models were built for both Ala and Lys using 1000 molecular geometries obtained from each of the four sampling approaches and were tested on 400 previously unseen (i.e., external and not trained for) molecular geometries obtained by the corresponding sampling approach. For example, kriging models built using geometries sampled using the PDB_NO_OPT method were tested on PDB_NO_OPT geometries, PDB/NM kriging models were tested on PDB/NM geometries, etc. Figure 6 shows the S‐curves for all four sampling methods. As an example of how to read such an S‐curve: 88% of geometries in the external test set for alanine's PDB_NO_OPT kriging models (top, red curve) have an error of maximum 4 kJ mol^−1^ (or 1 kcal mol^−1^) (where the red curve intersects the purple dashed line). The more the S‐curve is situated at the left of the plot, the more accurate the model that it describes. The error displayed by an S‐curve corresponds to that given by eq. (9), that is, $\left|\sum_{A,B}^{}E_{\text{AB}}^{\text{original}}-E_{\text{AB}}^{\text{predicted}}\right|$. As such, each point on an S‐curve corresponds to the absolute value of the sum of the errors of all predicted Coulombic interactions between pairs of atoms in one test molecular geometry, relative to the original interaction energies. This value is referred to as both the “total absolute error” and also the “S‐curve error.”

In connection with the information shown in Figure 6, note that Table 2 also reports the average absolute total error and the highest total error for each S‐curve. The alanine models built using PDB_OPT geometries (blue curve) had the lowest average error of 0.7 kJ mol^−1^. This is attributable to the lack of bond length and angle variation in the training and test sets and so the kriging problem is “less challenging” as there are fewer dimensions of conformational space being sampled. The second left‐most S‐curve corresponds to the predictions made using the models built using PDB_NO_OPT geometries (red curve). This is most likely a result of the lack of bond length variation of all hydrogen‐containing bonds. However, the PDB_NO_OPT does have the highest maximum total error of all sampling approaches, amounting to 25.8 kJ mol^−1^, despite the low average error. This is attributable to an alanine residue extracted from a crystal structure with a significantly stretched C_α_—C_β_ bond length and the H_α_—C_α_—C_β_ angle of 115°, which is significantly distorted from the stationary value of ∼108°. This fact illustrates the unsuitability of sampling amino acid geometries directly from crystal structures for QCTFF development, and emphasizes the need for a PDB/NM hybrid sampling approach. The kriging models obtained from the PDB/NM and NM sampled geometries perform worst overall, which is due to the large quantity of bond length sampling relative to the PDB_OPT and PDB_NO_OPT approaches. Despite being the S‐curves furthest to the right, PDB/NM and NM have average S‐curve errors of only 3.4 and 4.0 kJ mol^−1^, respectively. More than 60% of the test geometries of alanine were predicted by kriging models with an error of less than 1 kcal mol^−1^, a value often described as “chemical accuracy.”

**Figure 7:**
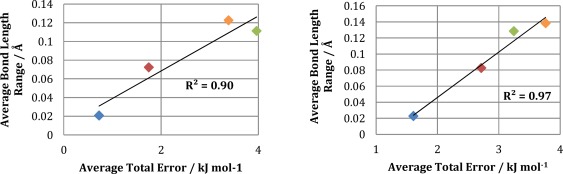
Average bond length deviation against average total (S‐curve) error for the different sampling approaches of Ala (left) and Lys (right): PDB_OPT (blue), PDB_NO_OPT (red), NM (green), and PDB/NM (orange). All data taken from Table 2. [Color figure can be viewed in the online issue, which is available at wileyonlinelibrary.com.]

It is interesting to note that the dihedral sampling appears to have less effect on the difficulty of the kriging problem than well‐sampled bond lengths. Figure 7 plots the average bond length range against average total (S‐curve) error for all four sampling approaches for Ala. The correlation between bond length and average S‐curve error ( $\frac{1}{N_{\text{train}}}\sum_{i=1}^{N_{\text{train}}}|\Delta E_{i,\text{system}}|$) is fairly strong, with an *R* ^2^ value of 0.90 (see Fig. 7). To illustrate this point further, the difference in average total error (S‐curve error or |Δ*E*_system_|) between PDB/NM and NM is 0.6 kJ mol^−1^ (see Table 2), although the PDB/NM approach samples a much larger range of dihedral conformational space than NM. In contrast to this, PDB_OPT, which has a much larger sampling of dihedral space than NM but also the smallest average range of bond lengths, has an average total error 3.3 kJ mol^−1^ lower than that of NM. This observation is a result of the following effect. Under the assumption of an identical dihedral sampling (as is the case for PDB_NO_OPT and PDB_OPT), increasing the range of bond lengths increases the volume of configurational space that the kriging models have to describe. This increase results in a more difficult kriging problem leading to increased prediction errors. It also is observed that changing a bond length has a dominant effect on the multipole moments of the atoms involved. This is illustrated in Supporting Information Figures S1–S3 where plots of C_α_ charge against both N—C_α_ bond length and backbone *ψ* angle are provided for the Ala geometries sampled by the PDB/NM, PDB_OPT and NM approaches, respectively. In both the PDB/NM and NM sampled plots, the C_α_ charge shows correlation with the N—C_α_ bond length but not with the *ψ* angle. It is only in the plots obtained from the PDB_OPT geometries (where the N—C_α_ bond length range is significantly reduced as a result of partial geometry relaxation) that any correlation between C_α_ charge and *ψ* can be seen. In summary, the correlation patterns above prove the dominance of bond length variation over dihedral sampling in posing a challenge to kriging.

The same conclusions may be drawn from the Lys S‐curves as from the Ala S‐curves: average bond length deviation is the most import factor dictating the average S‐curve error (Fig. 7), and although larger dihedral sampling increases the average error, it does this to a lesser extent than a large average bond length deviation. PDB_OPT has the lowest average S‐curve error (Lys: 1.6 kJ mol^−1^ and Ala: 0.7 kJ mol^−1^) due to the optimized bond lengths having the lowest average deviation (0.02 Å for both ALa and Lys). The PDB/NM S‐curve has the highest average error due to having the largest average bond length deviation and also a large dihedral sampling. PDB_NO_OPT has the largest maximum S‐curve error but, unlike the high error PDB_NO_OPT point on the Ala S‐curve, there is no clear structural reason behind the highest energy geometry. This could indicate that the geometry lies outside of the configurational space of the training set. The overall shape of an S‐curve may be related to the quality of the test geometries and the range of conformational space. For example, the NM S‐curve (green) is steep with only a small bend at the top. This is a result of the relatively small set of seed geometries causing the sampled geometries to be clustered close together. Therefore all test geometries are close to a training geometry within the kriging model and the errors remain constant throughout. In contrast, the PDB_NO_OPT (red) geometries are not clustered together and therefore the test geometries can be further away from the nearest training set geometry leading to larger errors. This gives rise to the less steep climb of this S‐curve and its longer tail toward the 100% ceiling.

**Figure 8:**
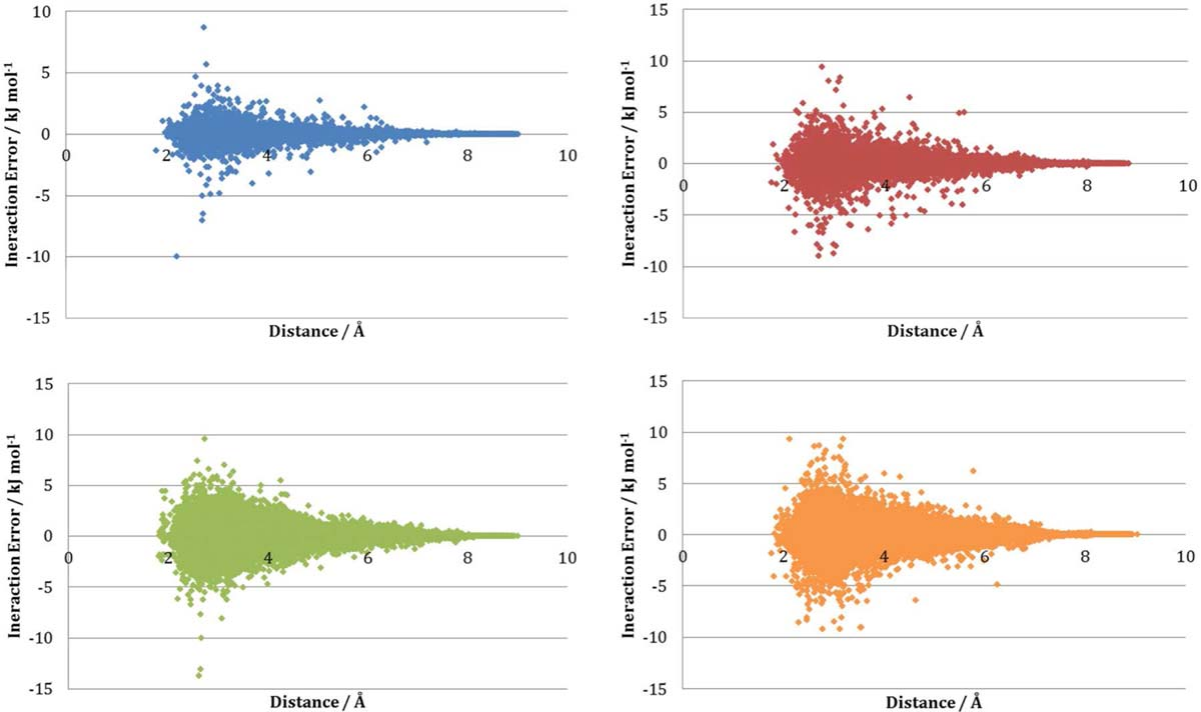
Individual intramolecular interaction prediction errors in Ala against interaction distance obtained for models built using the four sampling approaches: PDB_OPT (blue), PDB_NO_OPT (red), NM (green), and PDB/NM (orange). [Color figure can be viewed in the online issue, which is available at wileyonlinelibrary.com.]

Each point on the S‐curve is a sum of all 1,4 and higher intramolecular interaction prediction errors within a single test geometry ( $\left|\sum_{AB}\left(E_{AB}^{original}-E_{AB}^{predicted}\right)\right|$ from eq. (9)). Because of the sum, potential cancellation of positive and negative interaction errors is included within the S‐curve. To increase the transparency of the results we now focus on the construction of the S‐curve. Figure 8 shows all interaction errors for all Ala test geometries plotted against interaction distance for each sampling approach. The maximum absolute interaction error (max $\left|E_{AB}^{original}-E_{AB}^{predicted}\right|)\;$and average absolute interaction error (average $\left|E_{AB}^{original}-E_{AB}^{predicted}\right|$) for each approach is included in Table 2. Supporting Information Figure S4 shows a plot analogous to Figure 8 but for the sampled Lys geometries. The average absolute interaction errors follow the same trend as the total S‐curve error (PDB/NM ≈ NM > PDB_NO_OPT > PDB_OPT). For all sampling approaches used, the largest average absolute interaction error was only 0.4 kJ mol^−1^ (NM and PDB/NM sampled geometries). The correlation between average absolute interaction error and total error is very high with an *R* ^2^ of 0.97 for Ala and 0.99 for Lys. The plots of the average interaction prediction error versus the total error can be seen in Supporting Information Figure S5.

The standard deviation of the interaction errors for each method is provided in Table 3 for both Ala and Lys. Both PDB_OPT and PDB_NO_OPT have significantly larger standard deviations for Lys (0.5 kJ mol^−1^ and 0.8 kJ mol^−1^, respectively) than for Ala (0.2 kJ mol^−1^ and 0.4 kJ mol^−1^, respectively) as is expected by comparison of the blue and green plots in Figures 8 and Supporting Information S4. The PDB/NM interactions in Lys also have a larger standard deviation (0.7 kJ mol^−1^) than the PDB_NM interactions in Ala (0.6 kJ mol^−1^). Larger standard deviations emerge for Lys because it is a larger, more flexible molecule than Ala and so the kriging problem for PDB sampled geometries is much harder. Thus the kriging model is unable to find as good a solution for Lys than for Ala.

**Table 3:** Standard deviation of interaction prediction errors for both Ala and Lys from kriging models built from geometries sampled from the four sampling approaches (kJ mol^−1^).

Sampling	Ala	Lys
PDB_OPT	0.2	0.5
PDB_NO_OPT	0.4	0.8
NM	0.7	0.5
PDB/NM	0.6	0.7

### Optimum ratio of input geometries to sampled geometries for the PDB/NM sampling approach

The hybrid PDB/NM sampling approach has been presented as a means of sampling chemically relevant amino acid geometries for kriging models, taking advantage of the benefits afforded by both PDB and NM sampling whilst avoiding the problems associated with either method. The ratio (denoted 1:*n*) of PDB‐seed geometries (set to 1) to nonstationary NM sampled geometries (set to *n*) will now be discussed. The maximum dihedral sampling corresponds to a 1:1 ratio of PDB sampled “seed geometries” to NM sampled geometries. However, this ratio is computationally expensive because each PDB‐sampled amino acid seed geometry then needs to be partially geometry‐relaxed. Conversely, a ratio smaller than 1:1 (i.e., 1:*n* where *n*>1) requires fewer geometry optimizations, but decreases the sampling of (dihedral) conformational space. A smaller number of sampled geometries per PDB‐seed geometry will also affect the difficulty of the kriging problem as the sampling of conformational space will increase (assuming a constant training set size).

Training sets have been built, using the PDB/NM sampling approach, for ratios of seed geometries to NM‐sampled geometries of 1:20, 1:10, 1:4, 1:2, and 1:1, always with a total of 1200 NM‐sampled geometries in each case. These geometries were randomly reshuffled and then kriging models were built using 800 training geometries, and were tested on 400 (external) geometries.

**Figure 9:**
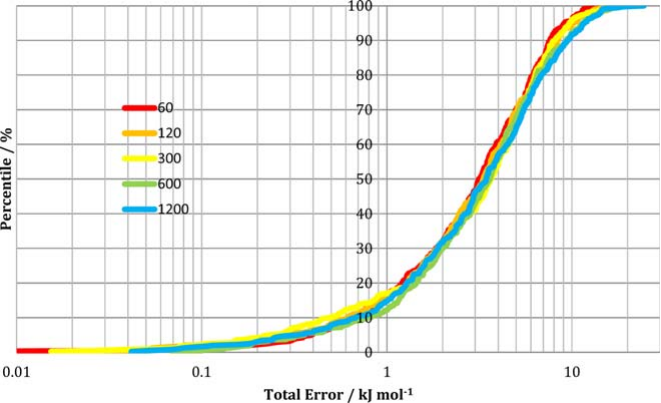
Errors in the predicted total 1–4 and higher electrostatic interaction energies of lysine by kriging models trained with molecular geometries obtained by the PDB/NM approach with different numbers of PDB‐seed geometries (see key on graph, 1200 corresponds to the 1:1 ratio in the main text). [Color figure can be viewed in the online issue, which is available at wileyonlinelibrary.com.]

**Figure 10:**
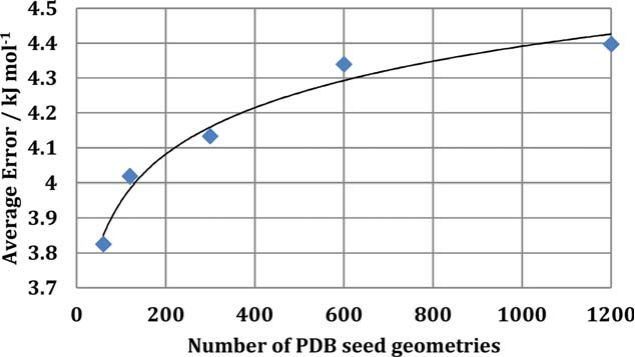
Average total error versus the number of PDB seed geometries for kriging models of lysine obtained from the PDB/NM sampling methodology. [Color figure can be viewed in the online issue, which is available at wileyonlinelibrary.com.]

Figure 9 shows the total energy S‐curve obtained for each training set. Increasing the number of PDB‐seed geometries does not significantly reduce the quality of the kriging model obtained. The average values of the S‐curve energies have been plotted against the number of input minima in Figure 10. There is a trend for a larger number of PDB‐seed geometries to have a higher average S‐curve error, but not dramatically so. The range of errors is only ∼0.6 kJ mol^−1^, between a 1:20 ratio of PDB‐seed geometries to sampled geometries (average error of 3.8 kJ mol^−1^) and a 1:1 ratio (average error of 4.4 kJ mol^−1^).

## Conclusions

The topological force field QCTFF contains a machine learning component that handles polarization and charge transfer (in a unified way). The machine learning method used, called kriging, needs a data set of molecular geometries to train on. Here we focus on obtaining a more realistic and relevant training set for amino acids. Before the current study, we sampled the training set by distorting the local energy minima of (peptide‐capped) amino acids (in the gas phase) according to NM obtained at those stationary points. Using the Protein Data Bank (PDB) we show here that these gas phase stationary points miss a number of important amino acid geometries that are present in a folded protein.

We present a new sampling approach that combines sampling of amino acid geometries from the Protein Data Bank (PDB) with nonstationary NM (NM) distortion. To the best of our knowledge the latter technique has not been attempted before. This hybrid approach is called PDB/NM and is tested on alanine and lysine, the most flexible amino acid of all. The use of the PDB greatly expands the sampling in the space of dihedral angles, both in range and density. Does this expansion lead to worse kriging models, given the larger variation and diversity in dihedral angles? The answer is negative because it turns out that the range in bond lengths is actually the prime factor in determining the difficulty and hence the predictive accuracy of the kriging models. As a result, the new PDB/NM sampling method (which is more “informed”) performs as well as the original “gas phase energy minimum” NM sampling. All kriging models lead to very good electrostatic energy prediction errors where more than 60% of external test geometries have a value of less than 4 kJ mol^−1^. Within the PDB/NM paradigm, the quality of the kriging models is not compromised much even if the training set consists of PDB‐sampled geometries only, which corresponds to maximum coverage of conformational space. In summary, the good news is that realistic dihedral angles can safely be combined with realistic bond lengths and angles into a single successful kriging model.

Further work utilizing rotamer libraries to guide the construction of training sets is planned to create training sets that do not depend on the crystal structures sampled from, but still mimic the structures expected in real proteins.

## Supporting information

Supporting Information
