# Supplementary material for: Realistic sampling of amino acid geometries for a multipolar polarizable force field
Source: J Comput Chem. 2015 Aug 3;36(24):1844–57. doi: 10.1002/jcc.24006 (PMC4973712; doi:10.1002/jcc.24006)
Supplement: Supplementary file 1 — Supporting Information [file JCC-36-1844-s001.docx]

**Supplementary Material**

## Timothy J. Hughes, Salvatore Cardamone, Paul L. A. Popelier*

**PART A**

List of 260 Protein Data Bank (PDB) files (*.pdb) from which the in-house script MOROS.pl sampled.

Two thousand amino acids were chosen from these files, by an average of almost 10 amino acids per PDB file.

| **1A70**  **1DP4**  **1KC1**  **1A53**  **1FL7**  **1MRU**  **1A99**  **1G0T**  **1MA3**  **1FT9**  **1KW8**  **1H6V**  **1ZOO**  **1QOL**  **2MSB**  **1B7V**  **1DFN**  **1GRE**  **1TYF**  **1CQP**  **1MIZ**  **1A5W**  **1IK3**  **1A2Z**  **1PM4**  **2BVW**  **1MXE**  **1AD1**  **1EAE**  **1JIZ**  **1FUJ**  **1F6Y**  **1HXY**  **1KEX**  **3PFL**  **1CR7**  **1JMO**  **1N8P**  **1A7Q**  **1GH7**  **1MA1**  **1JB6**  **1QKI**  **1RBC**  **1QB4**  **1MVB** | **1PRG**  **1RA4**  **1DIR**  **1F5W**  **1GN9**  **1A48**  **1IO1**  **1KJN**  **1DUV**  **1VAL**  **1FDR**  **1QMV**  **1M6B**  **1HMD**  **1CCD**  **1K0E**  **1HF2**  **1A7N**  **1CJ1**  **1MOL**  **1K2F**  **1IN5**  **1GOS**  **1GLJ**  **1DOV**  **1MTZ**  **1LJP**  **1B24**  **2DBV**  **1J54**  **1QF9**  **1KIU**  **1E9N**  **1VHH**  **1FC5**  **1QUS**  **1Q5Z**  **1AZO**  **1XWL**  **1DT6**  **1SZJ**  **1IHN**  **1HQ0**  **1K44**  **1IHO**  **1G88** | **1F9G**  **1YTI**  **1A22**  **1PYP**  **1GYV**  **1E0J**  **1IQA**  **1FC3**  **1PT7**  **1A6Q**  **1E6J**  **1FTX**  **1KKE**  **1B6C**  **1CSM**  **1D1P**  **1GOH**  **1FVR**  **1JH5**  **1QD9**  **1GCO**  **1I01**  **6PTD**  **1YDV**  **1VJS**  **1DNC**  **1CJQ**  **1A39**  **1RG7**  **1JV3**  **1M5U**  **1DM3**  **1F8R**  **1MHN**  **1LNS**  **1ATZ**  **1FG3**  **1SXB**  **1GQN**  **1M32**  **1QAG**  **1A0D**  **1LM7**  **1AY9**  **1I6I**  **1C8B** | **1K32**  **1RWR**  **1N2M**  **1CQK**  **2RAP**  **1B3Q**  **1FD4**  **1JLY**  **2ETA**  **1HYQ**  **1QB3**  **1ZXQ**  **1AZY**  **1SBP**  **1F6B**  **1GL0**  **1DLM**  **1HQN**  **1HCL**  **1QQC**  **1O8Q**  **1GMI**  **1QC7**  **2MHR**  **1QML**  **1E2E**  **1O70**  **1CJC**  **1FHE**  **1BOI**  **1HV8**  **1ILV**  **1SWA**  **1GQW**  **1TN3**  **1GR3**  **1H4R**  **1HBH**  **1KMM**  **1QF7**  **1BEE**  **1AYB**  **1EVQ**  **1PCZ**  **1M0Z**  **1DZJ** | **1GUZ**  **1G2V**  **1FAE**  **1L5X**  **1BF2**  **1HJ6**  **1BA1**  **1FEC**  **1GMG**  **1QQ2**  **1H0O**  **1GSE**  **1IK4**  **1PCF**  **1TRB**  **1A04**  **1I4W**  **1GMJ**  **1A0Z**  **1KXG**  **1IUG**  **1FCJ**  **1LNH**  **1REQ**  **1CJV**  **1Q8R**  **1EWF**  **1QIM**  **1CD0**  **1FC4**  **1I72**  **1RJ1**  **1QO4**  **1NUL**  **1JA3**  **1CLL**  **1TXX**  **1CII**  **1ML1**  **1HLG**  **1A00**  **1GEG**  **1LFK**  **1DFQ**  **1EH9**  **1IK6** | **1JZ0**  **1MVX**  **1AHP**  **2FHI**  **1TGJ**  **1K1O**  **1IJB**  **1L5Z**  **1A8O**  **1KAO**  **1MB0**  **1M3K**  **1B7D**  **1VCP**  **1FEB**  **1MMI**  **1EZX**  **1GMO**  **1DMH**  **1NYL**  **1JW9**  **3LYN**  **1JUQ**  **1KZQ**  **1K8T**  **1QFJ**  **1O88**  **1AGN**  **1B2P**  **1AYX** |
| --- | --- | --- | --- | --- | --- |

**Figure S1.** **PDB/NM approach:** dependence of Ala C_α_ charges (left) on N-C_α_ bond length and (right) on backbone ψ dihedral angle.

**Figure S2.** **PDB_OPT approach:** dependence of Ala C_α_ charges (left) on N-C_α_ bond length and (right) on backbone ψ dihedral angle.

**Figure S3.** **NM approach:** dependence of Ala C_α_ charges (left) on N-C_α_ bond length and (right) on backbone ψ dihedral angle.


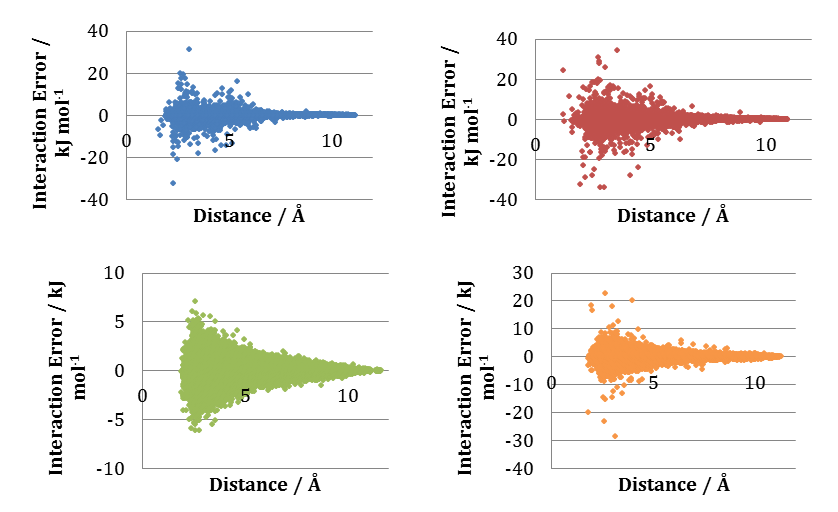


**Figure S4.** Individual intramolecular interaction prediction errors in Lys obtained for models built using the four sampling approaches: PDB_OPT (blue), PDB_NO_OPT (red), NM (green) and PDB/NM (orange).


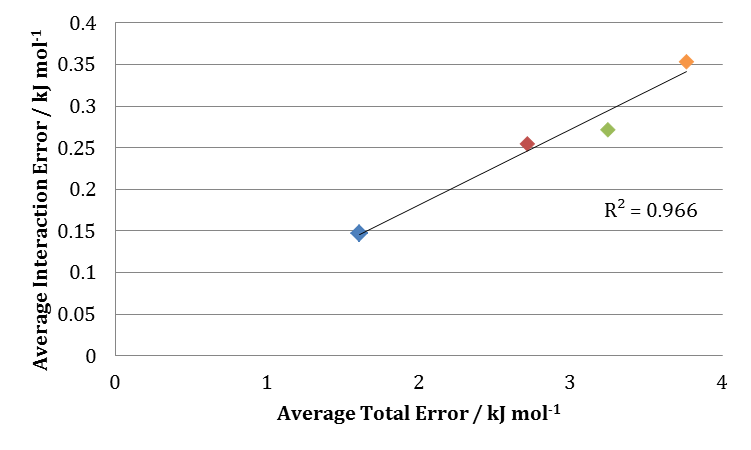

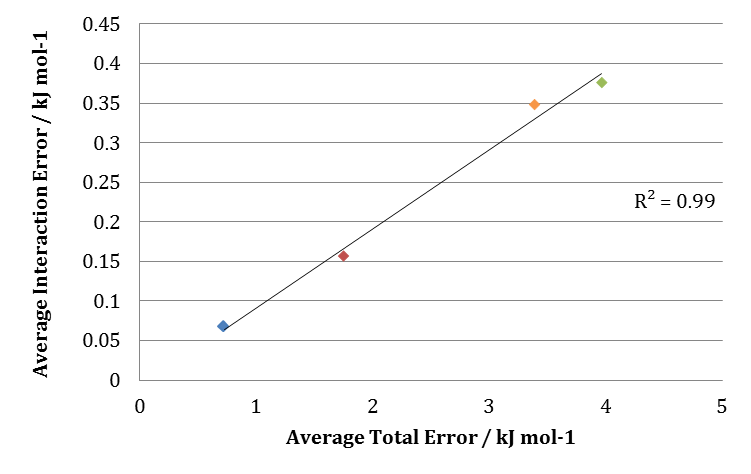


**Figure S5.** The average interaction energy prediction error versus average total (S-curve) error for Ala (left) and Lys (right) from kriging models trained with molecular geometries obtained by: PDB_OPT (blue), PDB_NO_OPT (red), NM (green) and PDB/NM (orange).

**PART B**

**Non-Stationary Point Normal Modes**

The normal modes for a molecular system are generally used as a dynamical basis, which is typically representative of the true vibrations of the system. However, the derivation of normal modes typically assumes a reference configuration that is situated at a stationary point on the potential energy surface. This assumption then appears to inhibit the derivation of normal modes at *non*-stationary point geometries. However, we show that a generalisation is actually straightforward, and involves the analysis of a second-order *inhomogeneous* differential equation.

**1 Overall Derivation**

**1.1 Kinetic Energy**

We begin with a molecular configuration, $\boldsymbol{x}=\left\{ x_{1},\ldots,x_{3N} \right\}$, and define a difference coordinate, $\Delta\boldsymbol{x}$, relative to some arbitrary configuration, $\boldsymbol{x}^{*}=\left\{ x_{1}^{*},\ldots,x_{3N}^{*} \right\}$, such that

| $\Delta\boldsymbol{x}=\boldsymbol{x-}\boldsymbol{x}^{\boldsymbol{*}}\boldsymbol{=}\left\{ x_{1}-x_{1}^{*},\ldots,x_{3N}-x_{3N}^{*} \right\}=\left\{ \Delta x_{1},\ldots,\Delta x_{3N} \right\}$ | (S1) |
| --- | --- |

We may do this without loss of generality for the following argument because we constrain $\boldsymbol{x}^{*}$ to be static. The classical expression for the kinetic energy of a system is then given by

| $T\left( \Delta\dot{x}_{1},\ldots,\Delta\dot{x}_{3N} \right)= \sum_{i}^{3N} \frac{m_{i}}{2}\left( \frac{d}{dt}\Delta x_{i} \right)^{2}$ | (S2) |
| --- | --- |

So far everything has been expressed in Cartesian coordinates but it is convenient to introduce mass-weighted (Cartesian) coordinates q_i_,

| $q_{i}=\Delta x_{i}\sqrt{m_{i}}$ | (S3) |
| --- | --- |

and substitution of its time derivative into eq S2, we obtain

| $T\left( \dot{q}_{1},\ldots,\dot{q}_{3N} \right)=\frac{1}{2}\sum_{i}^{3N} \left( \frac{d}{dt}q_{i} \right)^{2}=\frac{1}{2}\sum_{i}^{3N} \dot{q}_{i}^{2}$ | (S4) |
| --- | --- |

**1.2 Potential Energy**

The potential energy corresponding to a state, $V\left( \boldsymbol{x} \right)=V\left( x_{1},\ldots,x_{3N} \right)$ is given by a Taylor series about the predefined configuration $\boldsymbol{x}^{\boldsymbol{*}}$, leading to

| $2V\left( x_{1},\ldots,x_{3N} \right)=2V_{0}\left( x_{1}^{*},\ldots,x_{3N}^{*} \right)+ 2\sum_{i}^{3N} \left( x_{i}-x_{i}^{*} \right) \left. \frac{\partial V}{\partial x_{i}} \right\vert_{\boldsymbol{x}^{\boldsymbol{*}}}+ \sum_{i,j}^{3N} \left( x_{i}-x_{i}^{*} \right)\left( x_{j}-x_{j}^{*} \right)\left. \frac{\partial^{2}V}{\partial x_{i}\partial x_{j}} \right\vert_{\boldsymbol{x}^{\boldsymbol{*}}}+\ldots$ | (S5) |
| --- | --- |

The derivative factors are actually constants because they are evaluated at **x*** (a point to be kept in mind when differentiating further). Equation S6 introduces two definitions

| $\left. \frac{\partial V}{\partial x_{i}} \right\vert_{\boldsymbol{x}^{*}}=J_{i}^{'} \mathrm{and} \left. \frac{\partial^{2}V}{\partial x_{i}\partial x_{j}} \right\vert_{\boldsymbol{x}^{*}}=H_{ij}^{'}$ | (S6) |
| --- | --- |
| such that |  |
| $2V\left( x_{1},\ldots,x_{3N} \right)=2V_{0}\left( x_{1}^{*},\ldots,x_{3N}^{*} \right)+ 2\sum_{i}^{3N} \left( x_{i}-x_{i}^{*} \right) J_{i}^{'}+ \sum_{i,j}^{3N} \left( x_{i}-x_{i}^{*} \right)\left( x_{j}-x_{j}^{*} \right)H_{ij}^{'}+\ldots$ | (S7) |

The first-order and second-order spatial derivatives of the potential energy *V* correspond to elements of the Jacobian^[[1]](#footnote-1)^ and Hessian, respectively. By choosing $\boldsymbol{x}^{*}$ such that it occupies a stationary point on the potential energy surface, we are free to set $V\left( \boldsymbol{x}^{\boldsymbol{*}} \right)=0$. Additionally, the first derivative (Jacobian) term in the Taylor series necessarily goes to zero at this stationary point. By omitting all terms strictly higher than the second order, we obtain

| $2V\left( x_{1},\ldots,x_{3N} \right)= \sum_{i,j}^{3N} \left( x_{i}-x_{i}^{*} \right)\left( x_{j}-x_{j}^{*} \right)H_{ij}^{'}$ | (S8) |
| --- | --- |

It is useful to express the potential energy in the same coordinates as those used for the kinetic energy. This can be achieved using eq S3 and eq S9, which follows from eq S3,

| $\frac{\partial}{\partial q_{i}}=\frac{1}{\sqrt{m_{i}}}\frac{\partial}{\partial\left( x_{i}-x_{i}^{*} \right)}=\frac{1}{\sqrt{m_{i}}}\frac{\partial}{\partial x_{i}}$ | (S9) |
| --- | --- |

such that, when both substituted in eq S8 (and using eq S6), we obtain

| $2V\left( x_{1},\ldots,x_{3N} \right)= \sum_{i,j}^{3N} \Delta x_{i}\Delta x_{j}\left. \frac{\partial^{2}V}{\partial x_{i}\partial x_{j}} \right\vert_{\boldsymbol{x}^{*}}=\sum_{i,j}^{3N} {\frac{q_{i}q_{j}}{\sqrt{m_{i}m_{j}}}\sqrt{m_{i}m_{j}}}\left. \frac{\partial^{2}V}{\partial q_{i}\partial q_{j}} \right\vert_{\boldsymbol{0}}= \sum_{i,j}^{3N} H_{ij}q_{i}q_{j}=2V\left( q_{1},\ldots,q_{3N} \right)$ | (S10) |
| --- | --- |

where we hereafter call the mass-weighted elements of the Hessian, denoted $H_{ij}=\frac{1}{\sqrt{m_{i}m_{j}}}H_{ij}^{'}$ and $H_{ij}=\left. \frac{\partial^{2}V}{\partial q_{i}\partial q_{j}} \right|_{\boldsymbol{0}}\boldsymbol{=}\frac{1}{\sqrt{m_{i}m_{j}}}\left. \frac{\partial^{2}V}{\partial x_{i}\partial x_{j}} \right|_{\boldsymbol{x}^{\boldsymbol{*}}}$

**1.3 Equations of Motion**

Substituting eqs S4 and S10 into eq S11, which are the Euler-Lagrange equations of motion,

| $\frac{d}{dt}\frac{\partial T}{\partial\dot{q}_{k}}+\frac{\partial V}{\partial q_{k}}=0 \forall k=1,2,\ldots,3N$  leads to | (S11) |
| --- | --- |
| $\frac{d}{dt}\frac{\partial}{\partial\dot{q}_{k}} \left( \frac{1}{2}\sum_{i}^{3N} \dot{q}_{i}^{2} \right) + \frac{\partial}{\partial q_{k}}\left( \frac{1}{2}\sum_{i,j}^{3N} H_{ij}q_{i}q_{j} \right)=\frac{d}{dt}\left( \frac{1}{2}\sum_{i}^{3N} \frac{\partial}{\partial\dot{q}_{k}} \dot{q}_{i}^{2} \right) + \left( \frac{1}{2}\sum_{i,j}^{3N} {\frac{\partial}{\partial q_{k}}(H}_{ij}q_{i}q_{j}) \right)$ $=\frac{d}{dt}\left( \sum_{i}^{3N} \delta_{ik}\dot{q}_{i} \right)+\frac{1}{2}\sum_{i,j}^{3N} H_{ij}q_{i}\frac{\partial q_{j}}{\partial q_{k}}+\frac{1}{2}\sum_{i,j}^{3N} H_{ij}q_{j}\frac{\partial q_{i}}{\partial q_{k}}$ $=\frac{d\dot{q}_{k}}{dt}+\frac{1}{2}\sum_{i,j}^{3N} H_{ij}q_{i}\delta_{jk}+\frac{1}{2}\sum_{i,j}^{3N} H_{ij}q_{j}\delta_{ik}=\frac{d^{2}}{dt^{2}}q_{k}+\frac{1}{2}\sum_{i}^{3N} H_{ik}q_{i}+\frac{1}{2}\sum_{j}^{3N} H_{kj}q_{j}$ $=\frac{d^{2}}{dt^{2}}q_{k}+ \sum_{i}^{3N} H_{ik}q_{i}=0$ | (S12) |

where we have invoked the symmetric nature of the Hessian *H_ij_* = *H_ji_* and the fact that the last two sums are identical because *i* and *j* are dummy indices and therefore *j* can be written as *i*.

We have thus obtained a second-order homogeneous differential equation (HDE), the solution of which is a simple superposition of sinusoids of angular frequency $\omega$ and amplitudes $A_{k}$ and $B_{k}$ for the $k^{th}$ equation of motion,

| $q_{k}\left( t \right)=A_{k}\cos\left( \omega t \right)+ B_{k}\sin\left( \omega t \right)$ | (S13) |
| --- | --- |

We choose to use the more compact notation of a single sinusoid with a phase factor, $\phi$

| $q_{k}\left( t \right)=A_{k}\cos\left( \omega t+\phi\right)$ | (S14) |
| --- | --- |

Placing eq S14 into eq S12, we obtain

| $\frac{d^{2}}{dt^{2}}A_{k}\cos\left( \omega t+\phi\right)+\sum_{i}^{3N} H_{ik}A_{i}\cos\left( \omega t+\phi\right)=0$ | (S15) |
| --- | --- |
| $-\omega^{2}A_{k}\cos\left( \omega t+\phi\right)+ \sum_{i}^{3N} H_{ik}A_{i}\cos\left( \omega t+\phi\right)=0$ | (S16) |

The next step involves the cancellation of the factor $\cos\left( \omega t+\phi\right)$ in each term. However, this action places a constraint on the solution of eq S14, in case this factor is equal to zero, or when $\omega t+\phi=(2n+1)\pi/2$ where $n \in\mathbb{N}$. However, in that case we recover that $q_{k}\left( t \right)=0$ at the stationary point, which satisfies eq S16. Continuing with the case of non-zero $\cos\left( \omega t+\phi\right)$ we obtain

| $-\omega^{2}A_{k}+ \sum_{i}^{3N} H_{ik}A_{i}=0 and so\sum_{i}^{3N} A_{i}\left( H_{ik}- \omega^{2}\delta_{ik} \right)=0$ | (S17) |
| --- | --- |

This equation constitutes an eigensystem for which there exist $3N$ values of $\omega$, which give rise to non-trivial solutions for the $q_{k}\left( t \right)$, i.e. where $A_{k}\neq0$. These solutions may be found by diagonalisation of the mass-weighted Hessian, the eigenvalues of which correspond to the $3N$ frequencies, as may be seen by evaluation of the factor in parentheses in eq S17. Of course, this procedure is typically carried out in an internal coordinate basis, which renders six of the $3N$ degrees of freedom invariant. This then results in six of the eigenvalues of the mass-weighted Hessian being equal to zero, corresponding to the frequencies of the three global translational and three global rotational degrees of freedom. Note that, from here on, the index *k* runs from 1 to 3*N*-6, because we disregard those normal modes with a frequency of zero.

**2 General Derivation**

**2.1 Generalisation of the Potential Energy Expression**

It is clear from Section 1.2 that setting the first derivative of the potential to zero makes the derivation easier when considering the Euler-Lagrange equations of motion. We now assume that this first derivative does not vanish, and proceed with a general derivation for the equations of motion for a system that is not situated at a stationary point on the potential energy surface. We now rewrite eq S6 as follows,

| $2V\left( x_{1},\ldots,x_{3N} \right)= 2\sum_{i}^{3N} \left( x_{i}-x_{i}^{*} \right) J_{i}^{'}+ \sum_{i,j}^{3N} \left( x_{i}-x_{i}^{*} \right)\left( x_{j}-x_{j}^{*} \right)H_{ij}^{'}$ | (S18) |
| --- | --- |
| where we now use $\boldsymbol{x}^{\boldsymbol{*}}$ to denote the position vector of the system, which is not necessarily at a stationary point, and $J_{i}^{'}$ and $H_{ij}^{'}$ are still defined as in eq S6. Transformation to a mass-weighted coordinate basis,$\Delta x_{i}=$ $q_{i}/\sqrt{m_{i}} ,$ requires modification of the terms  $2V\left( q_{1},\ldots,q_{3N} \right)= 2\sum_{i}^{3N} {\frac{J_{i}^{'}}{\sqrt{m_{i}}}}q_{i}+ \sum_{i,j}^{3N} \frac{q_{i}q_{j}}{\sqrt{m_{i}m_{j}}} H_{ij}^{'}$ $2V\left( q_{1},\ldots,q_{3N} \right)= 2\sum_{i}^{3N} J_{i}q_{i}+ \sum_{i,j}^{3N} H_{ij}q_{i}q_{j}$ | (S19) |

where we have once again dropped the prime notation to denote the mass-weighted forms of the Jacobian and Hessian. How does the extra Jacobian term in eq S19, which is absent in eq S10, change the derivation in eq S12? Eq S20 focuses on the only term that requires revising, or

| $\frac{\partial V}{\partial q_{k}}=\frac{\partial}{\partial q_{k}}\left( \sum_{i}^{3N} J_{i}q_{i}+\frac{1}{2}\sum_{i,j}^{3N} H_{ij}q_{i}q_{j} \right)= \sum_{i}^{3N} \frac{\partial}{\partial q_{k}}{(J}_{i}q_{i})+ \frac{1}{2}\sum_{i,j}^{3N} \frac{\partial}{\partial q_{k}}{(H}_{ij}q_{i}q_{j})$ | (S20) |
| --- | --- |

The second term of eq S20 has previously been evaluated in eq S12. As such, we deal only with the first term

| $\sum_{i}^{3N} \frac{\partial}{\partial q_{k}}J_{i}q_{i}= \sum_{i}^{3N} \left[ J_{i}\frac{\partial}{\partial q_{k}}q_{i}+ q_{i}\frac{\partial}{\partial q_{k}}J_{i} \right]= \sum_{i}^{3N} J_{i}\delta_{ik}=J_{k}$ | (S21) |
| --- | --- |

where we have used the fact that *J_i_* is a constant value as can be derived from eq S6

| $J_{i}=\left. \frac{\partial V}{\partial q_{i}} \right\vert_{\boldsymbol{0}}=\frac{1}{\sqrt{m_{i}}}\left. \frac{\partial V}{\partial x_{i}} \right\vert_{\boldsymbol{x}^{\boldsymbol{*}}}\boldsymbol{=}\frac{1}{\sqrt{m_{i}}}J_{i}^{'}$ | (S22) |
| --- | --- |

such that eq S20 becomes

| $\frac{\partial V}{\partial q_{k}}= J_{k}+\sum_{i}^{3N} H_{ik}q_{i}$ | (S23) |
| --- | --- |

This new form of the first derivative of the potential V with respect to the mass-weighted coordinates then allows for a re-evaluation of the Euler-Lagrange equations of motion, yielding

| $\frac{d}{dt}\frac{\partial T}{\partial\dot{q}_{k}}+\frac{\partial V}{\partial q_{k}}= \frac{d^{2}}{dt^{2}}q_{k}+ J_{k}+\sum_{i}^{3N} H_{ik}q_{i}=0$ | (S24) |
| --- | --- |

which may be written in a form which mirrors eq S12, but with an inhomogeneous term at the right hand side

| $\frac{d^{2}}{dt^{2}}q_{k}+\sum_{i}^{3N} H_{ik}q_{i}=-J_{k}$  $\frac{d^{2}}{dt^{2}}q_{k}+\sum_{i}^{3N} H_{ik}q_{i}=F_{k}$ | (S25) |
| --- | --- |

where we have recognised that the negative of the mass-weighted Jacobian in this case is simply the mass-weighted force, i.e. $-J_{k}=F_{k}$.

**2.2 Inhomogeneous Differential Equation**

An inhomogeneous differential equation (IDE) is solved by the method of undetermined coefficients. To illustrate this methodology, we observe that the IDE of eq S25 is of the form

| $\frac{d^{2}}{dt^{2}}x+a_{0}x=f$ | (S26) |
| --- | --- |

Then, we form a solution by solving the underlying HDE, i.e.

| $\frac{d^{2}}{dt^{2}}x+a_{0}x=0$ | (S27) |
| --- | --- |

and simply append what is commonly termed a *particular solution*, $\xi$, dependent on the form of $f$. So, for our purposes, because the inhomogeneity associated with our differential equation takes the form of a constant, the particular solution then also takes the form of a constant. We then find a solution to eq S26 by inserting the particular solution, $\xi$, and so

| $\frac{d^{2}}{dt^{2}}\xi+a_{0}\xi=f$ $a_{0}\xi=f \therefore\xi=\frac{f}{a_{0}}$ | (S28) |
| --- | --- |

**2.3 Application**

We have previously shown that the equation of motion corresponding to the $k^{th}$ normal mode evaluated away from a stationary point on the potential energy surface is given by the solution to a second-order IDE, which we repeat here for convenience (i.e. eq S25),

| $\frac{d^{2}}{dt^{2}}q_{k}+\sum_{i}^{3N} H_{ik}q_{i}=F_{k}$ |  |
| --- | --- |

We proceed by noting that the solution to the underlying HDE has previously been evaluated in eq S14, which we reproduce here

| $q_{k}\left( t \right)=A_{k}\cos\left( \omega t+\phi\right)$ |  |
| --- | --- |

The inhomogeneous term in eq S25 is a constant, and so our particular solution will take the form of a constant, which we once again denote by $\xi_{k}$. Evaluating eq S25 with our particular solution as an argument

| $\frac{d^{2}}{dt^{2}}\xi_{k}+\sum_{i}^{3N} H_{ik}\xi_{k}=F_{k} \therefore\xi_{k}= \frac{F_{k}}{\sum_{i}^{3N} H_{ik}}$ | (S29) |
| --- | --- |

which leads to solution of our IDE

| $q_{k}\left( t \right)=A_{k}\cos\left( \omega t+\phi\right)+\frac{F_{k}}{\sum_{i}^{3N} H_{ik}}$ | (S30) |
| --- | --- |

By analysis, we note that eq S30 is dimensionally consistent, since $\xi_{k}$ has the form of a force divided by a force constant, which results in the dimension of length, as expected from the left hand side of eq S30. Additionally, we see that the above solution also satisfies the need for $q_{k}\left( t \right)$ to return to the form of eq S14 at a stationary point. In this case, $F_{k}=0$, which implies $\xi_{k}=0$, as required.

**PART C**

**Stochastic distribution versus equipartitioning of thermal energy**

Upon peer review of this manuscript, our decision to stochastically distribute the total thermal energy available to the system, $E=nk_{B}T/2$ through the modes has been queried. It has been suggested that assigning an energy of $k_{B}T/2$ to each mode, in keeping with equipartition, would be preferable. Indeed, given our “filtering” methodology (see Section 2.4), it was further suggested that increasing the temperature (thereby increasing the available thermal energy) could enhance conformational sampling.

Qualitatively, if a large amount of thermal energy is available to each mode, then there exists a high probability of “breaking” a bond or valence angle, as dictated by our filtering methodology. The breaking of a bond or valence angle leads to the rejection of a conformation, as the conformation is not physically valid. As such, the temperature, and consequently the thermal energy, must be lowered for the output of non-broken conformations. The maximum thermal energy, $E_{max}$, is therefore capped by the thermal energy required to perturb a bond or valence angle by an amount that exceeds the filtering criteria. Mathematically,

$E_{max}=\frac{nk_{B}T_{max}}{2}$ (S31)

such that

$\sqrt{\frac{2E_{max}}{nk_{B}}}\leq\left\{ \begin{matrix} l_{AB}k_{BOND} \\ \alpha_{0}k_{ANGLE} \end{matrix} \right. \forall l_{AB},\alpha_{0}$ (S32)

Thus, an arbitrary increase in temperature would be of little use given an equipartition of thermal energy, since there exists a well-defined limit to the temperature before the filtering methodology will reject all sampled conformations.

Given a stochastic distribution of the total thermal energy through the modes, $E_{max}$ can take on a more diverse set of values. This diversity stems from the non-negligible probability of assigning more energy into the more “conformationally flexible” modes without breaking a conformation, e.g. to modes that possess a large amount of torsional motion. As such, a stochastic distribution of the total thermal energy allows for less restrictive sampling of conformational space. We do, however, concede that a more thorough sampling of conformational space by the stochastic distribution of thermal energy through the modes is only guaranteed by a fortuitous distribution of the thermal energy, i.e. the assignment of the bulk of thermal energy to conformationally flexible modes. Therefore, we must make a distinction between, on one hand, the *capacity* to sample conformational space more thoroughly (which a stochastic distribution of energy offers), and, on the other hand, the *certainty* of a more rigorous sampling of conformational space (which is not guaranteed). The latter requires some degree of supervision by the user to ensure a desirable distribution of the energy. This supervision is currently performed by evaluating a file outputted by the in-house computer program TYCHE, yielding information on each redundant internal coordinate over the course of sampling (maximum and minimum values, range, etc.) Supervision can be automated somewhat by assigning a large portion of the total thermal energy for distribution through the lower frequency modes, which are typically more conformationally flexible. This “energetic biasing” into low frequency modes is planned to be implemented in the next version of TYCHE.

For comparative purposes, we have conducted a test to validate the logic presented above. In Figure A1, we present the evolution of the torsional angle of the R-group of histidine, given a stochastic distribution of thermal energy through the modes (green), and an equipartition of thermal energy through the modes (red). The temperature at which sampling was conducted was set to 400 K. The stochastic distribution of energy yields a slightly larger range of the torsional angle over the course of sampling. The RMSDs of the two sampling schemes differ by < 0.02 Å, presumably due to other degrees of freedom being sampled more thoroughly with the equipartition of thermal energy. As such, we conclude that *the stochastic distribution and equipartition of thermal energy yield similar results.*

However, a point which merits note is that the simulation time is an order of magnitude larger with the equipartition of thermal energy relative to the stochastic distribution. This is due to a significant deal more conformations which are filtered out with the former approach. To output non-broken conformations, the temperature was lowered slowly until valid conformations were actually sampled.


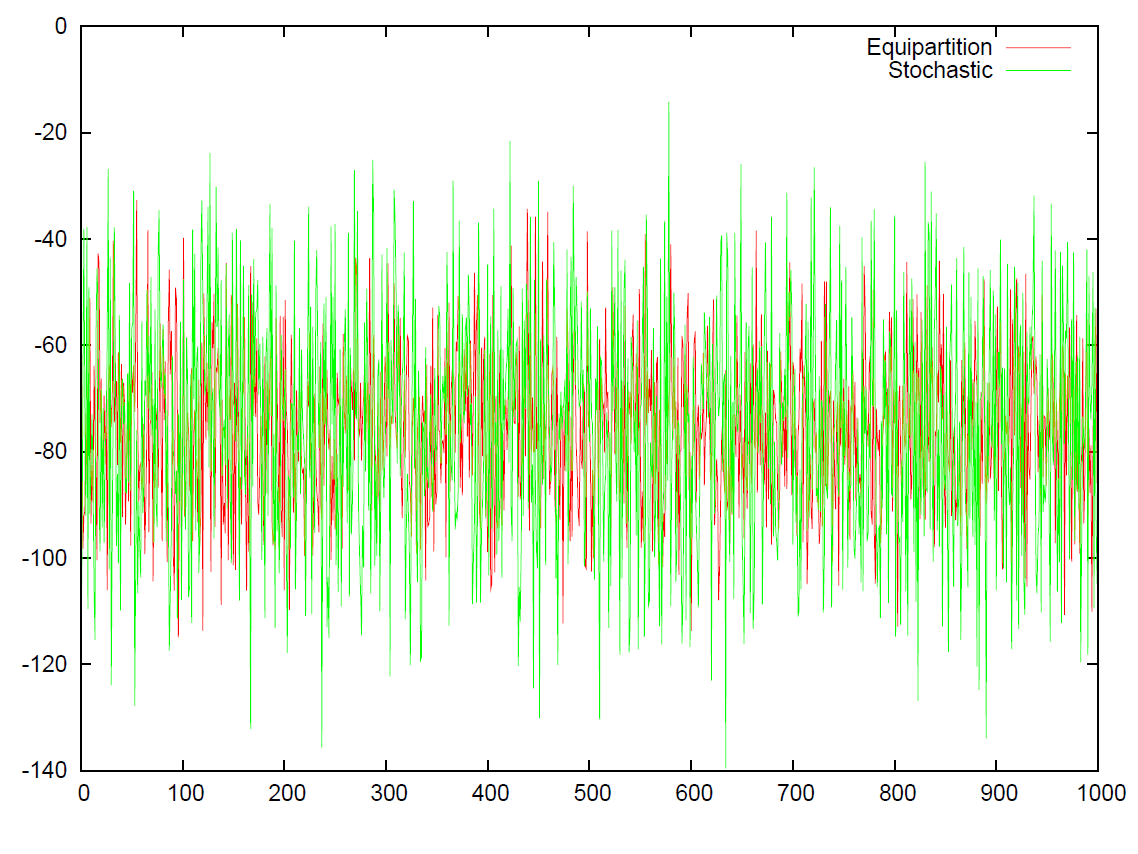


Dihedral Value

Frame Number

**Figure A1.** The evolution of the torsional angle of the R-group of histidine given a stochastic distribution (green) and an equipartition (red) of the total thermal energy available to a system at a temperature of 400 K.

1. The Jacobian $\boldsymbol{J}$ is defined as the derivative of the list of all first-order partial derivatives of a function $\boldsymbol{f}\boldsymbol{:}\mathbb{R}^{\boldsymbol{n}}\boldsymbol{\to}\mathbb{R}^{\boldsymbol{m}}$, with respect to those degrees of freedom, $\boldsymbol{x}$, over which $\boldsymbol{f}$ is defined. Taking the case of $m=1$, we see that $\boldsymbol{J}$ takes the form of $\left[ \partial f/\partial x_{1},\ldots,\partial f/\partial x_{n} \right]^{\top}$, which is the form used here. Of course, this list of (scalar) components is equivalent to the gradient of a scalar field, $\boldsymbol{\nabla}f$, but we prefer to work with its components. [↑](#footnote-ref-1)
